# Supplementary material for: Genetic components of human pain sensitivity: a protocol for a genome-wide association study of experimental pain in healthy volunteers
Source: BMJ Open. 2019 Apr 20;9(4):e025530. doi: 10.1136/bmjopen-2018-025530 (PMC6500241; doi:10.1136/bmjopen-2018-025530)
Supplement: Supplementary data [file bmjopen-2018-025530supp001.pdf]

Appendix 1: Demographic and Ancestry Questionnaire

1. Age (years):

2. Gender: female (0) male (1)

3. Do you consider yourself:

Black	
Mulatto	
Indigenous	
Moreno (Brown skin)	
Cobrizo (Coppery skin)	
Mestizo (Mixed)	
Pardo (Dark skin)	
Blanco (White skin)	
European	
Other (please describe in your own words)	

4. What ancestral proportions do you think you have? And in what proportions do you consider you have each of these components?

Mark with an X the percentage range you consider to have from each ancestry. If you believe you do not bear a specific ancestry, mark the range between 0-20%.

Indigenous		Black		European	
0-20% (none – very low)		0-20% (none – very low)		0-20% (none – very low)	
20-40% (low)		20-40% (low)		20-40% (low)	
40-60% (medium)		40-60% (medium)		40-60% (medium)	
60-80% (high)		60-80% (high)		60-80% (high)	
80-100% (very high - full)		80-100% (very high - full)		80-100% (very high - full)	

5. If you believe that you have another ancestral component (ancestors from other races), please let us know which ones:
