# Supplementary material for: Genetic components of human pain sensitivity: a protocol for a genome-wide association study of experimental pain in healthy volunteers
Source: BMJ Open. 2019 Apr 20;9(4):e025530. doi: 10.1136/bmjopen-2018-025530 (PMC6500241; doi:10.1136/bmjopen-2018-025530)
Supplement: Supplementary data [file bmjopen-2018-025530supp002.pdf]

Appendix 2: Current procedures for Quality Control and Imputation in CANDELA genotype data

The currently used protocol for quality control (QC) and genotype imputation for the CANDELA genotype data is quoted from the latest GWAS publication on this cohort[1]. The entire cohort comprises samples from five Latin American countries, thus being much bigger in size and more genetically diverse; in contrast, the current experimental pain cohort is smaller and restricted to one country, so some of the specific parameter choices could be slightly different while analysing its genotype data. However, the broad procedures and most parameters should remain the same.

Genotype data

DNA samples from participants were genotyped on the Illumina HumanOmniExpress chip, which includes 730,525 SNPs. PLINK v1.9[2] was used to exclude SNPs and individuals with more than 5% missing data, markers with minor allele frequency <1%, related individuals with IBD > 0.1 (i.e. removing 3rd degree relatives and higher), and those who failed the X-chromosome sex concordance check (sex estimated from X-chromosome heterozygosity not matching recorded sex information). After applying these filters, 669,462 SNPs and 6,357 individuals were retained for further analysis.

Due to the Native American, European and African admixture of the study sample, there is inflation in Hardy-Weinberg P-values. We therefore did not exclude markers based on Hardy-Weinberg deviation, but performed stringent quality controls at software and biological levels (see also Supplementary Figure 14 from Adhikari et al.[3]). The SNP quality metrics generated from the GenCall algorithm in GenomeStudio were used for quality control. SNPs with low GenTrain score (<0.7), low Cluster Separation score (<0.3) or high heterozygosity values (|het. excess|>0.5) were excluded[4]. The heterozygosity excess filter performs a function similar to a HWE check, but is more direct since it is

based on the heterozygosity value, which unlike the P value does not depend on sample size. Only SNPs that satisfy these criteria across all genotyping plates were retained[3].

The imputation 'concordance' score, which is a measure of poor genotyping quality, was also used to exclude some genotyped SNPs (see below). Finally, subsequent to the GWAS analyses (see below), the genotyping cluster plots for the index SNP identified were checked manually to verify genotyping quality.

An LD-pruned set of 160,858 autosomal SNPs was used to estimate continental ancestry using the ADMIXTURE program[5]. Genetic Principal Components (PCs) were also obtained from this LD-pruned subset of SNPs. Individual outliers, including individuals with >20% African or >5% East Asian ancestry, as estimated by ADMIXTURE, were removed. Outlier individuals observed on the PC scatter plots were also removed, and PCs were recalculated each time after the removal of such individuals until no outliers remained. The number of PCs to be included in the GWAS was determined by inspecting the proportion of variance explained and by checking scree and PC scatter plots.

Genotype imputation

The chip genotype data was phased using SHAPEIT2[6]. IMPUTE2[7] was then used to impute genotypes at untyped SNPs using variant positions from the 1000 Genomes Phase 3 data. The 1000 Genomes reference data set included haplotype information for 1,092 individuals across the world for 36,820,992 variant positions.

Positions that are monomorphic in 1000 Genomes Latin American samples (Colombia, Mexico and Puerto Rico) were excluded, leading to 11,025,002 SNPs being imputed in our dataset. Of these, 48,695 had imputation quality scores < 0.4 and were excluded. Median 'info' score (imputation certainty score) provided by IMPUTE2 for the remaining imputed SNPs was 0.986. The IMPUTE2 genotype probabilities at each locus were converted into most probable genotypes using PLINK v1.9[2]

(at the default setting of <0.1 uncertainty). Imputed SNPs with >5% uncalled genotypes or minor allele frequency < 1% were excluded.

IMPUTE2 provides a 'concordance' metric for chip genotyped SNPs, obtained by masking the SNP genotypes and imputing it using nearby chip SNPs. Genotyped SNPs having a low concordance value (< 0.7) or a large gap between info and concordance values (info_type0 – concord_type0 > 0.1), two suggested indicators of poor genotyping quality, were also removed. Median concordance values of the remaining chip SNPs was 0.994. After QC, the final imputed dataset contained genotypes for 9,143,600 SNPs.

References:

1. Adhikari, K. et al. A GWAS in Latin Americans highlights the convergent evolution of lighter skin pigmentation in Eurasia. Nat Commun 10, 358 (2019).
2. Chang, C.C. et al. Second-generation PLINK: rising to the challenge of larger and richer datasets. Gigascience 4, 7 (2015).
3. Adhikari, K. et al. A genome-wide association scan implicates DCHS2, RUNX2, GLI3, PAX1 and EDAR in human facial variation. Nat Commun 7, 11616 (2016).
4. Illumina Inc. GenomeStudio Genotyping Module v1.0 User Guide. (2008).
5. Alexander, D.H., Novembre, J. & Lange, K. Fast model-based estimation of ancestry in unrelated individuals. Genome Res 19, 1655-64 (2009).
6. O'Connell, J. et al. A general approach for haplotype phasing across the full spectrum of relatedness. PLoS Genet 10, e1004234 (2014).
7. Howie, B., Fuchsberger, C., Stephens, M., Marchini, J. & Abecasis, G.R. Fast and accurate genotype imputation in genome-wide association studies through pre-phasing. Nat Genet 44, 955-9 (2012).
