# Supplementary material for: Genetic components of human pain sensitivity: a protocol for a genome-wide association study of experimental pain in healthy volunteers
Source: BMJ Open. 2019 Apr 20;9(4):e025530. doi: 10.1136/bmjopen-2018-025530 (PMC6500241; doi:10.1136/bmjopen-2018-025530)
Supplement: Supplementary data [file bmjopen-2018-025530supp003.pdf]

Appendix 3: Power Calculations

The power for Genome-wide association studies of experimental pain phenotypes for varying sample and effect sizes was estimated following the formulae described in Visscher et al[1]. Estimated power is shown for a range of effect sizes for experimental pain phenotypes taken from existing experimental pain studies. The statistical software R version 3.4.1[2] was used to perform the calculations and produce the figures. The codes are published on https://github.com/kaustubhad/gwas-power.

In whole-genome SNP-based GWAS studies, the association analysis is usually conducted with a multivariate linear regression model, where the trait values are regressed onto a SNP genotype (with additive coding) and other covariates which commonly include age, gender, BMI, and genetic principal components (PCs). The p-value threshold[1,3] for genome-wide significant associations is commonly 5×10^{-8}, while the threshold for a suggestive significant association is commonly 10^{-5}.

Under the commonly used GWAS linear regression model, the term corresponding to the SNP genotype leads to a test statistic which is distributed as a chi-square distribution with 1 degrees of freedom (df). Under the null of no association it is a central chi-square, whereas under the alternative it is a non-central chi-square distribution whose non-centrality parameter (NCP) can be derived[1,4]. Power of a GWAS depends on the allele frequency of the SNPs through their effect on the NCP.

The significance threshold for the test statistic under a central chi-square (i.e. under the null) of df 1 and p-value cut-off $p = 5\times10^{-8}$ is:

$$t = F^{-1}(1 - p, 1) = 29.72$$

Where F is the cumulative distribution function (CDF) of a central chi-square distribution, i.e. t is the (1-p)-th quantile of the distribution[4].

The power (P) of detecting an association with a trait in a GWAS, which is the probability of the observed test statistic exceeding the significance threshold t under the alternative, depends on its non-centrality parameter (λ)[4].

$$P = 1 - G(t, \lambda, 1)$$

Where G is the CDF of the non-central chi-square distribution, and df = 1 as usual.

The non-centrality parameter (λ) depends on sample size (n) and the proportion of phenotypic variance that is explained by the SNP[1], denoted by q^2.

$$\lambda = n \times \frac{q^2}{1 - q^2}$$

Thus we can vary the values of sample size (n) and the proportion of phenotypic variance explained by the SNP, denoted by (q^2), to obtain various values λ of and calculate the corresponding power (P).

These derivations include various model assumptions, e.g. the chi-square distribution assumption depends on the trait being continuous and the errors being approximately normally distributed. The assumption of normal distribution of errors might not hold in reality but in large sample sizes commonly used in GWAS they tend to hold approximately.

Similar calculations can be performed for the commonly used p-value threshold for suggestive significance, $p = 10^{-5}$. The significance threshold for the chi-square test statistic at a suggestive level is t = 19.51.

References:

1. Visscher PM, Wray NR, Zhang Q, et al. 10 Years of GWAS Discovery: Biology, Function, and Translation. Am J Hum Genet 2017;101(1):5-22. doi: 10.1016/j.ajhg.2017.06.005

2. R Core Team. R: A language and environment for statistical computing. Vienna, Austria2018 [Available from: https://www.R-project.org/2018.

3. Adhikari K, Reales G, Smith AJ, et al. A genome-wide association study identifies multiple loci for variation in human ear morphology. Nature communications 2015;6:7500. doi: 10.1038/ncomms8500

4. Rao CR. Linear Statistical Inference and its Applications. 2nd ed: Wiley 1973.
