# Supplementary material for: Genetic components of human pain sensitivity: a protocol for a genome-wide association study of experimental pain in healthy volunteers
Source: BMJ Open. 2019 Apr 20;9(4):e025530. doi: 10.1136/bmjopen-2018-025530 (PMC6500241; doi:10.1136/bmjopen-2018-025530)
Supplement: Author's manuscript [file bmjopen-2018-025530.draft_revisions.pdf]

BMJ Open is committed to open peer review. As part of this commitment we make the peer review history of every article we publish publicly available.

When an article is published we post the peer reviewers' comments and the authors' responses online. We also post the versions of the paper that were used during peer review. These are the versions that the peer review comments apply to.

The versions of the paper that follow are the versions that were submitted during the peer review process. They are not the versions of record or the final published versions. They should not be cited or distributed as the published version of this manuscript.

BMJ Open is an open access journal and the full, final, typeset and author-corrected version of record of the manuscript is available on our site with no access controls, subscription charges or pay-per-view fees (http://bmjopen.bmj.com).

If you have any questions on BMJ Open's open peer review process please email editorial.bmjopen@bmj.com

BMJ Open

Genetic components of human pain sensibility: an experimental pain study

Journal:	*BMJ Open*
Manuscript ID	bmjopen-2018-025530
Article Type:	Protocol
Date Submitted by the Author:	24-Jul-2018
Complete List of Authors:	Schmid, Annina B.; Oxford University, Nuffield Department of Clincial Neurosciences<br>Adhikari, Kaustubh; University College London, London, UK, Department of Genetics, Evolution and Environment; University College London, Department of Cell and Developmental Biology<br>Ramirez-Aristeguieta, Luis Miguel; Universidad de Antioquia, QST Lab. Faculty of Odontology<br>Chacón-Duque, Juan-Camilo; university College London, Department of Genetics, Evolution and Environment<br>Poletti, Giovanni; Universidad Peruana Cayetano Heredia, Unidad de Neurobiologia Molecular y Genética, Laboratorios de Investigación y Desarrollo, Facultad de Ciencias y Filosofía<br>Gallo, Carla; Universidad Peruana Cayetano Heredia, Unidad de Neurobiologia Molecular y Genética, Laboratorios de Investigación y Desarrollo, Facultad de Ciencias y Filosofía<br>Rothhammer, Francisco; Tarapacá University, Instituto de Alta Investigación<br>Bedoya, Gabriel; Universidad de Antioquia, GENMOL (Genética Molecular)<br>Ruiz-Linares, Andres; Fudan University, Ministry of Education Key Laboratory of Contemporary Anthropology and Collaborative Innovation Center of Genetics and Development, School of Life Sciences and Human Phenome Institute; Aix-Marseille Université, CNRS, EFS, ADES<br>Bennett, David; Oxford University, Nuffield Department of Clincial Neurosciences
Keywords:	GENETICS, nociception, pain sensitivity, pain, genome wide association study, hyperalgesia

SCHOLARONE™
Manuscripts

Genetic components of human pain sensibility: an experimental pain study

Annina B Schmid[1*], Kaustubh Adhikari[2,3*], Luis-Miguel Ramirez-Aristeguieta[4], Juan-Camilo Chacón-Duque[2], Giovanni Poletti[5], Carla Gallo[5], Francisco Rothhammer[6], Gabriel Bedoya[7], Andres Ruiz-Linares[8,9], David L Bennett[1]

1 Nuffield Department of Clinical Neurosciences, Oxford University, Oxford, UK

2 Department of Genetics, Evolution and Environment, University College London, London, UK

3 Department of Cell and Developmental Biology, University College London, London, UK

4 QST Lab. Faculty of Odontology, University of Antioquia, Medellin, Colombia

5Unidad de Neurobiologia Molecular y Genética, Laboratorios de Investigación y Desarrollo, Facultad de Ciencias y Filosofía, Universidad Peruana Cayetano Heredia, Lima, Peru

6 Instituto de Alta Investigación, Tarapacá University, Chile;

7 GENMOL (Genética Molecular), Universidad de Antioquia, Medellín, Colombia

8 Ministry of Education Key Laboratory of Contemporary Anthropology and Collaborative Innovation Center of Genetics and Development, School of Life Sciences and Human Phenome Institute, Fudan University, Shanghai, China.

9 Aix-Marseille Université, CNRS, EFS, ADES, Marseille, France

* authors contributed equally to this work

Corresponding authors

David LH Bennett

Oxford University, Nuffield Department of Clincial Neurosciences

For peer review only

For peer review only - http://bmjopen.bmj.com/site/about/guidelines.xhtml

John Radcliffe Hospital

West Wing Level 6

OX39DU Oxford

United Kingdom

Phone: +44 (0)1865 231512

Andrés Ruiz-Linares

Ministry of Education Key Laboratory of Contemporary Anthropology and Collaborative

Innovation Center of Genetics and Development

Fudan University

2005#Songhu Road, Yangpu District

Shanghai 200438, China

86-21-51630503

Corresponding authors email addresses

David.bennett@ndcn.ox.ac.uk

andresruiz@fudan.edu.cn

Word count 3410

Keywords: genetics, pain, nociception, pain sensitivity, hyperalgesia, genome wide association study

For peer review only

Abstract:

Introduction: Pain constitutes a major component of the global burden of diseases. Recent studies suggest a strong genetic contribution to pain susceptibility and severity. Whereas most of the available evidence relies on candidate gene association or linkage studies, research on the genetic basis of pain sensitivity using genome-wide association studies (GWAS) is still in its infancy. This protocol describes a GWAS on genetic contributions to baseline pain sensitivity and nociceptive sensitization in a sample of unrelated healthy individuals of mixed European/Native American/Sub-Saharan African ancestry.

Methods and Analysis: A genome wide association study (GWAS) on genetic contributions to pain sensitivity in the naïve state and following nociceptive sensitization will be conducted in unrelated healthy individuals of mixed ancestry. Mechanical and thermal pain sensitivity will be evaluated with a battery of quantitative sensory tests evaluating pain thresholds. In addition, variation in mechanical and thermal sensitisation following topical application of mustard oil to the skin will be evaluated.

Ethics and Dissemination: This study received ethical approval from the University College London research ethics committee (3352/001) and from the bioethics committee of the Odontology Faculty at the University of Antioquia (CONCEPTO 01-2013). Findings will be disseminated to commissioners, clinicians and service users via papers and presentations at international conferences.

For peer review only

Article Summary

Strengths and Limitations of this study

- We propose a genome-wide association study of both baseline pain sensitivity as well as nociceptive sensitisation.
- The study will be conducted in an admixed population in Colombia, with variable proportions of European, Native American and Sub-Saharan African ancestry
- Phenotypic data will be collected by a single trained examiner, thus producing high quality measures.
- Because we are focusing on highly reproducible pain phenotypes obtained at a single centre, our initial sample size is limited to 1500-2000 participants. This will allow us to identify only genetic variants that have intermediate and large (but not small) effect sizes.

For peer review only

INTRODUCTION

Pain constitutes a major component of the global burden of diseases with low back and neck pain representing the single leading cause for years lived with disability followed closely by migraine and other musculoskeletal disorders[1]. Pain is a multidimensional experience involving a highly complex interaction of physical, biochemical, physiological, cognitive, emotional, behavioural and sociocultural factors. A growing number of studies in patient populations suggest that genetics is an important contributory factor to pain susceptibility and severity[2-4]. Of note, twin studies using either clinical pain outcomes or experimental pain models suggest that sensitivity to pain has a heritability of up to 55%[5-10]. Interestingly, the heritability varies greatly depending on the clinical pain outcome or the sensory modality tested in experimental pain models[7]. There is evidence that different sensory modalities may have distinct genetic components contributing to their variance in humans,[10] and this is consistent with animal models which underline the distinct neurobiology mediating different sensory modalities[11].

A powerful technology for identifying genetic determinants of human complex phenotypes are genome wide association studies (GWAS). However, GWAS analyses of pain remain limited, mainly due to the high numbers of individuals required to enable adequate power, and the complexities of accurately phenotyping traits that ultimately represent a personal subjective perception[2]. In contrast to disease cohorts where pain variation may be influenced by the severity of the disease process or its treatment, experimental pain studies measuring baseline pain sensitivity have the advantage of studying one stimulus in a standard condition (e.g., controlling intensity, location and stimulus duration). To date, several candidate gene studies have been performed to determine genetic influences on human pain sensitivity using experimental pain models[5-9 12 13], however, to our knowledge, a full GWAS has not yet been

reported. One genome-wide study evaluated association for SNPs revealed by exome sequencing in a sub-set of a twins cohort that were identified as having particularly high or low heat pain sensitivity. Using pathway analysis there was significant enrichment for variants in genes of the angiotensin pathway[14]. Although stimuli are given in controlled conditions, there is evidence that findings from experimental pain models can be predictive of clinically relevant pathological pain such as post-operative pain[15]. An as yet unanswered question is, whether specific experimental pain measures may be particularly predictive of clinically relevant pain. This therefore was a motivation for us to include a number of sensory modalities in our protocol.

An important drawback of most genetic studies in the pain field so far is that they have been performed mostly in populations of European ancestry, thus they have explored only a small fraction of human phenotypic and genetic diversity[16 17]. This is important in the study of pain as recent studies point to variable pain thresholds for European Americans, African Americans and Latinos[18-20]. However, it is not clear, if this variation in pain thresholds relates to differences in neurobiological mechanisms or other factures such as social or cultural factors. In addition, recent studies suggest that a phenotype associated with increased sensitization of the nociceptive system due to temporal summation may render people vulnerable to developing clinically relevant pain[21]. However, most studies focus on pain phenotyping in the naïve state rather than sensitized state; only a handful of studies have investigated a genetic component underlying nociceptive sensitization[7 22]. We therefore intend to use an algogen (mustard oil, an agonist of the ligand gated ion channel TRPA1) in order to sensitise the nociceptive system to replicate the changes that could occur in pathological pain states.

For peer review only

This protocol proposes a GWAS on genetic contributions to baseline pain sensitivity and nociceptive sensitization in a sample of unrelated healthy individuals of mixed European/Native American/African ancestry. We will evaluate baseline cutaneous pain thresholds as well as the variation in sensitisation following mustard oil application, a controlled model of tissue injury. Elucidating the genetic basis of pain variation has the potential to reveal targets for future analgesic development. This can be translated into improved pain management potentially tailored to an individual's pain risk or resilience, including sensitivity differences between different human populations.

METHODS AND ANALYSIS:

Participants

Healthy participants aged 18-40 will be recruited in Medellin, Colombia via public noticeboards at local Universities, distribution of flyers and through the local print media. In addition, we are inviting previous participants from the CANDELA GWAS (Consortium for the Analysis of the Diversity and Evolution of Latin America)[23] to participate in this project. Participants will be excluded if they have chronic pain or any chronic medical condition (e.g, diabetes, neurodegenerative, musculoskeletal or psychiatric conditions). Participants currently taking analgesics, anti-inflammatories, opioids, antihistamines, antidepressants or anti-epilectic drugs will be excluded. Women who are pregnant or in their menstrual phase (self-report) will be excluded from the study. Participants will be advised to not smoke or consume coffee within 1 hour of testing and to avoid psycho-active substances or alcohol within 8 hours prior to testing. Further exclusion criteria include current or past self-inflicted

For peer review only - http://bmjopen.bmj.com/site/about/guidelines.xhtml

injuries, dermatomal, traumatic or infectious conditions affecting the arm and a history of severe allergic reactions to any kind of medication, materials, food or insect bites.

Procedure

Participants will attend a single appointment in the quantitative sensory testing laboratory at the Universidad de Antioquia, Medellín. Following informed consent demographic and clinical data will be collected (e.g., age, gender, BMI, general health, blood pressure). Participants will also complete the validated Spanish versions of the Hamilton Anxiety Rating Scale[24] and the Quick Inventory of Depressive Symptomatology (QIDS-SR16)[25]. Participants will also answer questions related to their ancestry (e.g., self-perceived skin colour, estimated percentage of their ancestry (e.g., indigenous, black, european).

Evaluation of sensory function in the naïve state

The procedure for assessment of sensory function is detailed in Figure 1. We will determine sensory function in the naïve state and following nociceptive sensitization. Baseline sensory function will be evaluated using specific static and dynamic quantitative sensory tests. These include cold and warm detection thresholds, thermal sensory limen and heat pain thresholds using a thermotester (Q-sense, Medoc, Israel, 30x30mm thermode size). Recording of thermal thresholds will strictly follow published quantitative sensory testing guidelines[26]. Mechanical pain thresholds will be evaluated using a 20 piece von Frey hair set (Touch Test, North Coast, USA) wich exerts differening forces (9.8, 13.7, 19.6, 39.2, 58.8, 78.5, 98.1, 147.1, 255.0, 588.4, 980.7, 1765.2, 2942.0 mN). The von Frey hairs will be applied at a rate of 2s on, 2s off in ascending order starting from 9.8mN baseline stimulus until participants first perceive the stimulus as sharp (pricking). Subsequently, the hairs will be applied in descending order until the stimuls is perceived as blunt. The geometric mean of five series of

For peer review only - http://bmjopen.bmj.com/site/about/guidelines.xhtml

ascending and descending stimuli is defined as the mechanical pain threshold. Wind-up ratio will be determined with numerical pain ratings in a visual analogue scale (VAS 0-100) for a single stimulus followed by the average pain rating for a train of 10 stimuli applied at 1 Hz within the same $1cm^2$ using a 255mN von Frey hair. This will be repeated five times and the ratio will be established as the mean rating of the trains of stimuli divided by the mean rating of the single stimuli. Vibration detection thresholds will be determined by recording the mean of 3 disappearance thresholds with a Rydel-Seiffer tuning fork. Pressure pain thresholds will be recorded in triplicate with a manual algometer (Wagner Instruments, USA) and their mean used for analysis.

The side to be tested will be randomized and patients will first be familiarized with the sensory tests on the forearm on the control side, before performing the actual measurements on the test arm. All tests will be performed half way over the volar side of the forearm except for VDT (ulnar styloid) and PPT (thenar muscles).

Mustard oil evoked nociceptive sensitisation

After the baseline sensory measures, an acetate template will be used to mark a star with eight spokes each containing eight points at 1cm increments on the volar forearm (Figure 2). The skin temperature will be standardised by placing the 32° C warm thermode on the volar forearm for 5 minutes before starting. We will then apply a sensitization paradigm using mustard oil (Allyl isothiacyanate, Sigma) as previously performed[27]. Mustard oil activates the ion channel TRPA1 and evokes skin flare and nociceptive sensitization[28]. A small cotton swab soaked in 30% mustard oil diluted in olive oil will be applied to a 0.64 cm^2 area on the volar forearm and held in place with a tegaderm (3M) for 10 minutes[27]. During this time, pain

For peer review only - http://bmjopen.bmj.com/site/about/guidelines.xhtml

scores will be recorded every 30 seconds using an electronic visual analogue scale ranging from 0 to 100. After 10 minutes, the mustard oil will be removed and the area of the skin flare will be recorded to the nearest 0.5 cm at each spoke[7]. Eight triangular shapes will be created by joining the points on adjacent spokes and the total area will be calculated by adding all triangular segments. The area of mustard oil application will be subtracted from the total area to determine the area of secondary flare.

After mapping of the flare, the area of brush-evoked hypersensitivity will be determined with a brush (Nr 5 Senselab, Somedic, Sweden) by applying 1 cm long strokes at each of the points on the eight spokes, starting from the outside and moving towards the sensitized centre. The area of punctuate hypersensitivity will be determined with a 98.1mN filament (Bailey Instruments, UK) following the same procedure[27]. As for the flare, the primary area of mustard oil application will be subtracted from both hypersensitive areas such that the recorded areas represent secondary hyperalgesia/hypersensitivity.

Following mustard oil sensitization, the mechanical and heat pain thresholds will be repeated using the same methods as described above. All post-sensitisation tests will be performed within 5 minutes of mustard oil removal.

Reliability of naïve and sensitized sensory function protocol

To determine intratester reliability, we repeated the sensory function protocol performed by the same investigator in n=12 healthy volunteers on two different occasions within 2-6 weeks. Intraclass correlation coefficients (ICC 3.1) revealed good to excellent agreement for all sensory testing variables (Table 1).

For peer review only

Genotyping

Each participant will donate blood or saliva (Oragene OG-500, Genotek, Canada) for DNA extraction. DNA samples will be genotyped on the Illumina HumanOmniExpress chip containing ~700,000 markers. In volunteers who already participated in the CANDELA GWAS, genotype data is already available and will be reused.

Whole-genome genotype data from the Illumina array will undergo quality control[29] to exclude any markers or samples that fail stringent thresholds. Quality metrics provided by the genotype calling algorithm in the Illumina GenomeStudio software[30], such as the GenTrain score, cluster separation score, and excess heterozygosity rates will be used to filter poorly genotyped SNPs. Subsequent SNP- and sample-level quality control thresholds such as missingness will be applied. Sex mismatch between records and genetic data of X & Y chromosomes will be checked. Only samples and SNPs that pass all criteria will be retained for analysis.

Statistical analysis

Sample size calculation

The power for Genome-wide association studies of experimental pain phenotypes for varying sample and effect sizes was estimated following the formulae described in Visscher et al[31]. Estimated power is shown for a range of effect sizes for experimental pain phenotypes taken from existing experimental pain studies and the statistical software R version 3.4.1[32] was used to perform the calculations and produce the figures. The codes are published on https://github.com/kaustubhad/gwas-power.

For peer review only - http://bmjopen.bmj.com/site/about/guidelines.xhtml

In whole-genome SNP-based GWAS studies, the p-value threshold for genome-wide significant associations is commonly 5×10^{-8} [31 33], which is calculated under a multivariate linear regression model, where the trait values are regressed onto a SNP genotype (with additive coding) and other covariates which commonly include age, gender, BMI, and genetic principal components (PCs). Under such a linear regression model, the term corresponding to the SNP genotype leads to a test statistic which is distributed as a chi-square distribution with 1 degrees of freedom (df). Under the null of no association it is a central chi-square, whereas under the alternative it is a non-central chi-square distribution whose non-centrality parameter (NCP) can be derived[31 34].

The significance threshold for the test statistic under a central chi-square (i.e. under the null) of df 1 and p-value cut-off $p = 5\times10^{-8}$ is:

$$t = F^{-1}(1 - p, 1) = 29.72$$

Where F is the cumulative distribution function (CDF) of a central chi-square distribution, i.e. t is the (1-p)-th quantile of the distribution[34].

The power (P) of detecting an association with a trait in a GWAS, which is the probability of the observed test statistic exceeding the significance threshold t under the alternative, depends on its non-centrality parameter (λ)[34].

$$P = 1 - G(t, \lambda, 1)$$

Where G is the CDF of the non-central chi-square distribution, and df = 1 as usual. The non-centrality parameter (λ) depends on sample size (n) and the proportion of phenotypic variance that is explained by the SNP, denoted by q^2[31].

$$\lambda = n \times \frac{q^2}{1 - q^2}$$

For peer review only - http://bmjopen.bmj.com/site/about/guidelines.xhtml

Thus we can vary the values of sample size (n) and the proportion of phenotypic variance explained by the SNP, denoted by (q^2), to obtain various values λ of and calculate the corresponding power (P).

These derivations include various model assumptions, e.g. the chi-square distribution assumption depends on the trait being continuous and the errors being approximately normally distributed. The assumption of normal distribution of errors might not hold in reality but in large sample sizes commonly used in GWAS they tend to hold approximately. Similar calculations can be performed for the commonly used p-value threshold for suggestive significance, $p = 10^{-5}$. The significance threshold for the chi-square test statistic at a suggestive level is t = 19.51.

Figure 3A shows estimated power (in percentage) under the standard GWAS settings of using whole-genome genotyping data and a p-value significance threshold of 5×10^{-8}. Sample size (n) varies from 100 to 5000, while the proportion of trait variance explained by the marker (q2, in percentage) varies from 0.01% to 6%. As sample size increases, power increases quickly for a range of trait variance values to reach 100%.

Figure 3B shows estimated power (in percentage) under the same settings but a suggestive p-value significance threshold of 10^{-5}. As expected, power is higher at similar sample and effect sizes for this less stringent threshold.

The range of trait variance has been taken from Doehring et al[35], which provides estimates for the proportion of trait variance explained by a SNP for several experimental pain phenotypes and multiple markers. The values ranged from 0.02% to 6%. Some of the traits

For peer review only - http://bmjopen.bmj.com/site/about/guidelines.xhtml

were the same as the traits investigated here, while some other traits were different. Nevertheless, the distributions of trait variance for the two groups of traits are very similar, as seen in Figure 4A.

Power of a GWAS depends on the allele frequency of the SNPs through their effect on the NCP. While the majority of GWAS studies are conducted in European-origin individuals, including the experimental pain study used to determine sample size here[35], our population of interest is an admixed Latin American population. Therefore, we wanted to assess the distribution of allele frequencies in Europeans versus Colombians for SNPs studied for or associated with experimental pain in various studies[35 36]. Minor allele frequencies were obtained for all such reported SNPs from the 1000 Genomes project database[16] for Western Europeans (from Britain (GBR), Utah residents from Northern & Western Europe (CEU), Spain (IBS), and Tuscany in Italy (TSI)) and Colombians (from Medellin in Colombia, CLM, where this study will be performed). Allele frequency distributions for both Europeans and Colombians are shown in Figure 4B. The two distributions are quite similar, with the Colombian distribution slightly more spread out. This is somewhat expected as the Colombians have on average 60% European (Spanish) ancestry[23]. Having a well spread out distribution of allele frequencies is important in a GWAS as low-frequency alleles have lower power for a given sample and effect size[37]. Here, the comparison to European allele frequency distribution suggests that the current Colombian cohort will have nearly equivalent power to any European-based cohort. In contrast to European-only cohorts though, our cohort will have the advantage that alleles present in other continental populations such as Sub-Saharan Africans or Native Americans that are not present in Europeans would also be detectable in a GWAS, and could be followed up in replication cohorts of specific ethnicities.

For peer review only - http://bmjopen.bmj.com/site/about/guidelines.xhtml

The CANDELA project includes genotpyes of ~2000 patients from Medellin. We anticipate to contact and phenotype 50-75% of these participants as well as contacting an additional 500 participants to bring the initial sample to 1500-2000.

Data analysis plan

The cleaned genetic data will first be merged with reference samples worldwide, such as the 1000 Genomes Project[16], Simons Genome Diversity Project[38], Estonian Biocentre Human Genome Diversity Panel[39], and additional European and Native American samples that are particularly relevant for Latin American populations[40]. The merged dataset will be checked for genetic outliers, through genetic principal components (PCs) and continental ancestry proportions (using supervised Admixture[41]), and for unexpected genetic similarities. These steps can often detect any sample misplacement or contamination, which might be reflected in sex mismatch, unexpected genetic similarities, or inflated heterozygosity rates[29 42].

The genetic data allows estimation of the narrow-sense heritability of any quantitative trait, which is the fraction of trait variance that is explained by the genetic data. Estimates of heritability, obtained using the software GCTA[43] will provide an idea of which traits have more of a biological basis versus which are more environmentally determined, and thus which traits would be more amenable to genetic analysis for discovery of associated genetic variants.

To facilitate better identification of such loci, the genotype data will be imputed to approximately 10 million loci using the 1000 Genomes Phase 3 imputation reference panel[44] by first haplotype phasing using SHAPEIT2[45] and then imputation using Impute2[46]. Quality

For peer review only - http://bmjopen.bmj.com/site/about/guidelines.xhtml

control of the imputed genotypes will be performed using recommended thresholds on imputation quality score, concordance metrics, and proportion of high-probability calls.

GWAS studies will be conducted in Plink2[47] to perform single-locus association studies for any trait across the whole genome in an additive multivariate linear regression model[31]. Covariates will be used in the regression to adjust for any other sources of trait variability, such as basic variables like age and sex, and genetic principal components will be used to control for population substructure[44]. An extension of this regression model which better controls for any cryptic relatedness or population substructure, called the mixed linear mode analysis (MLMA), will also be performed in GCTA[43]. These single-locus association results, obtained as P-values, will be visualized via the manhattan plot. Commonly used P-value thresholds for selecting associated loci are 5×10^{-8} for genome-wide significance and 10^{-5} for suggestive significance[31].

There are several extensions of the single-trait single-locus association studies that increase power for detecting associated loci: combining several related traits that may share a biological basis, using multivariate Wald tests as implemented in MultiPhen[48]; or gene-based tests that combine signals across all loci in a gene to increase signal strength and reduce the burden of multiple testing, such as set-based models implemented in Plink2[47] or fastBAT implemented in GCTA[49]. These might help detect additional loci that are under-powered in classical GWAS due to smaller effect sizes.

For peer review only

Patient and Public Involvement

No patient involvement is performed during this study.

Ethics and dissemination:

This study received ethical approval from the University College London research ethics committee (3352/001) and from the bioethics committee of the Odontology Faculty at the University of Antioquia (CONCEPTO 01-2013). Findings will be disseminated to commissioners, clinicians and service users via papers and presentations at international conferences such as the biennial World Congress of International Association for the Study of Pain. We will also add data to the publicly available painnetworks.org database.

Author Contributions:

DLB and ARL conceptualised the study. Funding was acquired by DLB, ABS, GB and ARL. ABS, JCCD, KA, and LMR have contributed to the study methodology. The first draft of the protocol was prepared by ABS, DLB, KA and ARL and all authors provided critical evaluation and approved the final manuscript.

Funding statement:

Work leading to this publication was funded by grants from: the Newton Fund Institutional Links Grant from the British Research Council (grant number 216398412), the Excellence Initiative of Aix-Marseille University - A*MIDEX (a French "Investissements d'Avenir" programme), and from a Wellcome Trust Senior Scientist Fellowship to DLB (ref. no. 095698z/11/z and 202747/Z/16/Z). KA was supported by a Wellcome Investigator Award WT107055AIA (to Prof. C.D. Stern). ABS was supported by the National Institute for Health Research (NIHR) Biomedical Research Centre (BRC) Oxford. The views expressed are those of the author(s) and not necessarily those of the NHS, the NIHR or the Department of Health.

For peer review only

Competing interests statement

The authors declare not competing interest.

For peer review only

REFERENCES:

1. Disease GBD, Injury I, Prevalence C. Global, regional, and national incidence, prevalence, and years lived with disability for 310 diseases and injuries, 1990-2015: a systematic analysis for the Global Burden of Disease Study 2015. *Lancet* 2016;388(10053):1545-602. doi: 10.1016/S0140-6736(16)31678-6

2. Zorina-Lichtenwalter K, Meloto CB, Khoury S, et al. Genetic predictors of human chronic pain conditions. *Neuroscience* 2016;338:36-62. doi: 10.1016/j.neuroscience.2016.04.041

3. Bennett DL, Woods CG. Painful and painless channelopathies. *Lancet Neurol* 2014;13(6):587-99. doi: 10.1016/S1474-4422(14)70024-9

4. Denk F, McMahon SB. Neurobiological basis for pain vulnerability: why me? *Pain* 2017;158 Suppl 1:S108-S14. doi: 10.1097/j.pain.0000000000000858

5. Belfer I, Diatchenko L. Pain Genetics: basic to translational science. Oxford: Wiley 2014.

6. Nielsen CS, Stubhaug A, Price DD, et al. Individual differences in pain sensitivity: genetic and environmental contributions. *Pain* 2008;136(1-2):21-9. doi: 10.1016/j.pain.2007.06.008 [published Online First: 2007/08/19]

7. Norbury TA, MacGregor AJ, Urwin J, et al. Heritability of responses to painful stimuli in women: a classical twin study. *Brain* 2007;130(Pt 11):3041-9. doi: 10.1093/brain/awm233

8. Hocking LJ, Generation S, Morris AD, et al. Heritability of chronic pain in 2195 extended families. *European journal of pain* 2012;16(7):1053-63. doi: 10.1002/j.1532-2149.2011.00095.x [published Online First: 2012/02/18]

9. Angst MS, Phillips NG, Drover DR, et al. Pain sensitivity and opioid analgesia: a pharmacogenomic twin study. *Pain* 2012;153(7):1397-409. doi: 10.1016/j.pain.2012.02.022 [published Online First: 2012/03/27]

For peer review only - http://bmjopen.bmj.com/site/about/guidelines.xhtml

10. Nielsen CS, Knudsen GP, Steingrimsdottir OA. Twin studies of pain. *Clin Genet* 2012;82(4):331-40. doi: 10.1111/j.1399-0004.2012.01938.x

11. Mogil JS. Pain genetics: past, present and future. *Trends Genet* 2012;28(6):258-66. doi: 10.1016/j.tig.2012.02.004

12. Kim H, Mittal DP, Iadarola MJ, et al. Genetic predictors for acute experimental cold and heat pain sensitivity in humans. *J Med Genet* 2006;43(8):e40. doi: 10.1136/jmg.2005.036079

13. Kim H, Neubert JK, San Miguel A, et al. Genetic influence on variability in human acute experimental pain sensitivity associated with gender, ethnicity and psychological temperament. *Pain* 2004;109(3):488-96. doi: 10.1016/j.pain.2004.02.027

14. Williams FM, Scollen S, Cao D, et al. Genes contributing to pain sensitivity in the normal population: an exome sequencing study. *PLoS Genet* 2012;8(12):e1003095. doi: 10.1371/journal.pgen.1003095

15. Werner MU, Mjobo HN, Nielsen PR, et al. Prediction of postoperative pain: a systematic review of predictive experimental pain studies. *Anesthesiology* 2010;112(6):1494-502. doi: 10.1097/ALN.0b013e3181dcd5a0

16. Genomes Project C, Auton A, Brooks LD, et al. A global reference for human genetic variation. *Nature* 2015;526(7571):68-74. doi: 10.1038/nature15393

17. Popejoy AB, Fullerton SM. Genomics is failing on diversity. *Nature* 2016;538(7624):161-64. doi: 10.1038/538161a

18. Rahim-Williams FB, Riley JL, 3rd, Herrera D, et al. Ethnic identity predicts experimental pain sensitivity in African Americans and Hispanics. *Pain* 2007;129(1-2):177-84. doi: 10.1016/j.pain.2006.12.016 [published Online First: 2007/02/14]

For peer review only

19. Rahim-Williams B, Riley JL, 3rd, Williams AK, et al. A quantitative review of ethnic group differences in experimental pain response: do biology, psychology, and culture matter? *Pain Med* 2012;13(4):522-40. doi: 10.1111/j.1526-4637.2012.01336.x

20. Bagwath Persad LA, Kamerman PR, Wadley AL. Predictors of Cold and Pressure Pain Tolerance in Healthy South African Adults. *Pain Med* 2017;18(11):2126-37. doi: 10.1093/pm/pnw291

21. Weissman-Fogel I, Granovsky Y, Crispel Y, et al. Enhanced presurgical pain temporal summation response predicts post-thoracotomy pain intensity during the acute postoperative phase. *J Pain* 2009;10(6):628-36. doi: 10.1016/j.jpain.2008.12.009

22. Tegeder I, Adolph J, Schmidt H, et al. Reduced hyperalgesia in homozygous carriers of a GTP cyclohydrolase 1 haplotype. *Eur J Pain* 2008;12(8):1069-77. doi: 10.1016/j.ejpain.2008.02.004

23. Ruiz-Linares A, Adhikari K, Acuna-Alonzo V, et al. Admixture in Latin America: geographic structure, phenotypic diversity and self-perception of ancestry based on 7,342 individuals. *PLoS Genet* 2014;10(9):e1004572. doi: 10.1371/journal.pgen.1004572

24. Hamilton M. The assessment of anxiety states by rating. *Br J Med Psychol* 1959;32(1):50-5.

25. Rush AJ, Trivedi MH, Ibrahim HM, et al. The 16-Item Quick Inventory of Depressive Symptomatology (QIDS), clinician rating (QIDS-C), and self-report (QIDS-SR): a psychometric evaluation in patients with chronic major depression. *Biol Psychiatry* 2003;54(5):573-83.

26. Rolke R, Magerl W, Campbell KA, et al. Quantitative sensory testing: a comprehensive protocol for clinical trials. *Eur J Pain* 2006;10(1):77-88. doi: 10.1016/j.ejpain.2005.02.003

For peer review only

For peer review only - http://bmjopen.bmj.com/site/about/guidelines.xhtml

27. Kremeyer B, Lopera F, Cox JJ, et al. A gain-of-function mutation in TRPA1 causes familial episodic pain syndrome. *Neuron* 2010;66(5):671-80. doi: 10.1016/j.neuron.2010.04.030

28. Koltzenburg M, Lundberg LE, Torebjork HE. Dynamic and static components of mechanical hyperalgesia in human hairy skin. *Pain* 1992;51(2):207-19.

29. Anderson CA, Pettersson FH, Clarke GM, et al. Data quality control in genetic case-control association studies. *Nat Protoc* 2010;5(9):1564-73. doi: 10.1038/nprot.2010.116

30. Guo Y, He J, Zhao S, et al. Illumina human exome genotyping array clustering and quality control. *Nat Protoc* 2014;9(11):2643-62. doi: 10.1038/nprot.2014.174

31. Visscher PM, Wray NR, Zhang Q, et al. 10 Years of GWAS Discovery: Biology, Function, and Translation. *Am J Hum Genet* 2017;101(1):5-22. doi: 10.1016/j.ajhg.2017.06.005

32. TEam RC. R: A language and environment for statistical computing. Vienna, Austria2018 [Available from: https://www.r-project.org/2018.

33. Adhikari K, Reales G, Smith AJ, et al. A genome-wide association study identifies multiple loci for variation in human ear morphology. *Nature communications* 2015;6:7500. doi: 10.1038/ncomms8500

34. CR R. Linear Statistical Inference and its Applications. 2nd ed: Wiley 1973.

35. Doehring A, Kusener N, Fluhr K, et al. Effect sizes in experimental pain produced by gender, genetic variants and sensitization procedures. *PLoS ONE* 2011;6(3):e17724. doi: 10.1371/journal.pone.0017724

36. Duan G, Guo S, Zhang Y, et al. The Effect of SCN9A Variation on Basal Pain Sensitivity in the General Population: An Experimental Study in Young Women. *J Pain* 2015;16(10):971-80. doi: 10.1016/j.jpain.2015.06.011

For peer review only

37. NM L, Lange C. The Fundamentals of Modern Statistical Genetics: Springer 2011.

38. Mallick S, Li H, Lipson M, et al. The Simons Genome Diversity Project: 300 genomes from 142 diverse populations. *Nature* 2016;538(7624):201-06. doi: 10.1038/nature18964

39. Pagani L, Lawson DJ, Jagoda E, et al. Genomic analyses inform on migration events during the peopling of Eurasia. *Nature* 2016;538(7624):238-42. doi: 10.1038/nature19792

40. Reich D, Patterson N, Campbell D, et al. Reconstructing Native American population history. *Nature* 2012;488(7411):370-4. doi: 10.1038/nature11258

41. Alexander DH, Novembre J, Lange K. Fast model-based estimation of ancestry in unrelated individuals. *Genome Res* 2009;19(9):1655-64. doi: 10.1101/gr.094052.109

42. Chacon-Duque JC, Adhikari K, Fuentes-Guajardo M, et al. Latin Americans show wide-spread Converso ancestry and the imprint of local Native ancestry on physical appearance. *bioRxiv* 2018 doi: 10.1101/252155

43. Yang J, Lee SH, Goddard ME, et al. GCTA: A Tool for Genome-wide Complex Trait Analysis. *The American Journal of Human Genetics*;88(1):76-82. doi: 10.1016/j.ajhg.2010.11.011

44. Adhikari K, Fuentes-Guajardo M, Quinto-Sanchez M, et al. A genome-wide association scan implicates DCHS2, RUNX2, GLI3, PAX1 and EDAR in human facial variation. *Nature communications* 2016;7:11616. doi: 10.1038/ncomms11616

45. O'Connell J, Gurdasani D, Delaneau O, et al. A general approach for haplotype phasing across the full spectrum of relatedness. *PLoS Genet* 2014;10(4):e1004234. doi: 10.1371/journal.pgen.1004234

For peer review only - http://bmjopen.bmj.com/site/about/guidelines.xhtml

46. Howie B, Fuchsberger C, Stephens M, et al. Fast and accurate genotype imputation in genome-wide association studies through pre-phasing. *Nat Genet* 2012;44(8):955-9. doi: 10.1038/ng.2354

47. Chang CC, Chow CC, Tellier LC, et al. Second-generation PLINK: rising to the challenge of larger and richer datasets. *Gigascience* 2015;4:7. doi: 10.1186/s13742-015-0047-8

48. O'Reilly PF, Hoggart CJ, Pomyen Y, et al. MultiPhen: joint model of multiple phenotypes can increase discovery in GWAS. *PLoS ONE* 2012;7(5):e34861. doi: 10.1371/journal.pone.0034861

49. Bakshi A, Zhu Z, Vinkhuyzen AA, et al. Fast set-based association analysis using summary data from GWAS identifies novel gene loci for human complex traits. *Sci Rep* 2016;6:32894. doi: 10.1038/srep32894

For peer review only

For peer review only - http://bmjopen.bmj.com/site/about/guidelines.xhtml

Table 1: Intratester reliability of sensory function protocol

	ICC(3,1)	95% CI	p-value
CDT	0.728	0.277, 0.914	0.003
WDT	0.764	0.351, 0.927	<0.0001
TSL	0.638	0.161, 0.878	0.005
HPT	0.752	0.339, 0.922	0.002
MPT	0.928	0.767, 0.979	<0.0001
WUR	0.634	0.113, 0.880	0.012
VDT	0.956	0.860, 0.987	<0.0001
PPT	0.734	0.305, 0.915	0.002
VAS	0.893	0.667, 0.970	<0.0001
Flare area	0.610	0.095, 0.869	0.015
Brush-evoked allodynia	0.756	0.365, 0.922	0.001
Punctuate hyperalgesia	0.615	0.094, 0.871	0.013
Post-sensitisation MPT	0.941	0.808, 0.983	<0.0001
Post-sensitisation HPT	0.758	0.339, 0.924	0.002

CDT: cold detection threshold, WDT: warm detection threshold, TSL: thermal sensory limen, HPT: heat pain threshold, MPT: mechanical pain threshold, WUR: windup ratio, VDT: vibration detection threshold, PPT: pressure pain threshold, VAS: visual analogue scale

For peer review only

Figure legends

Figure 1: Study procedure. The graph demonstrates the study procedure which starts with familiarisation of the participants with QST, followed by baseline sensory function measurements. Mustard oil (AITC) will subsequently be applied for 5 minutes. After removal of mustard oil, a pain VAS will be taken every 30 seconds for 10 minutes. Subsequently, the nociceptive sensitisation measurements will be taken.

CDT, WDT: cold, warm detection thresholds; TSL: thermal sensory limen; VDT: vibration detection threshold; MPT: mechanical pain threshold; HPT: heat pain threshold; PPT: pressure pain threshold; WUR: windup ratio

Figure 2: Method to determine area of flare, punctuate hyperalgesia and allodynia.

Figure 3: Estimated power. (A) estimated power (in percentage) setting the significance threshold at $5x10^{-8}$, the commonly used threshold for genome-wide significance in GWAS studies. (B) estimated power with the significance threshold set at 10^{-5}, the commonly used threshold for suggestive significance.

The X-axis denotes a range of sample sizes (n) in a GWAS, the Y-axis represents the proportion of trait variance (q^2) explained by a causal marker. Power of detecting the causal marker at a specific (n, q^2) combination is represented by a color gradient. Contour lines for power at 10% intervals are also shown.

Figure 4: (A) Comparable distributions of trait variance explained by a single marker from Doehring et al[35] for traits included in our study and those not included. (B) Allele frequency

For peer review only

For peer review only - http://bmjopen.bmj.com/site/about/guidelines.xhtml

distributions of associated loci for Europeans and Colombians taken from previously published cohorts.

For peer review only

For peer review only - http://bmjopen.bmj.com/site/about/guidelines.xhtml

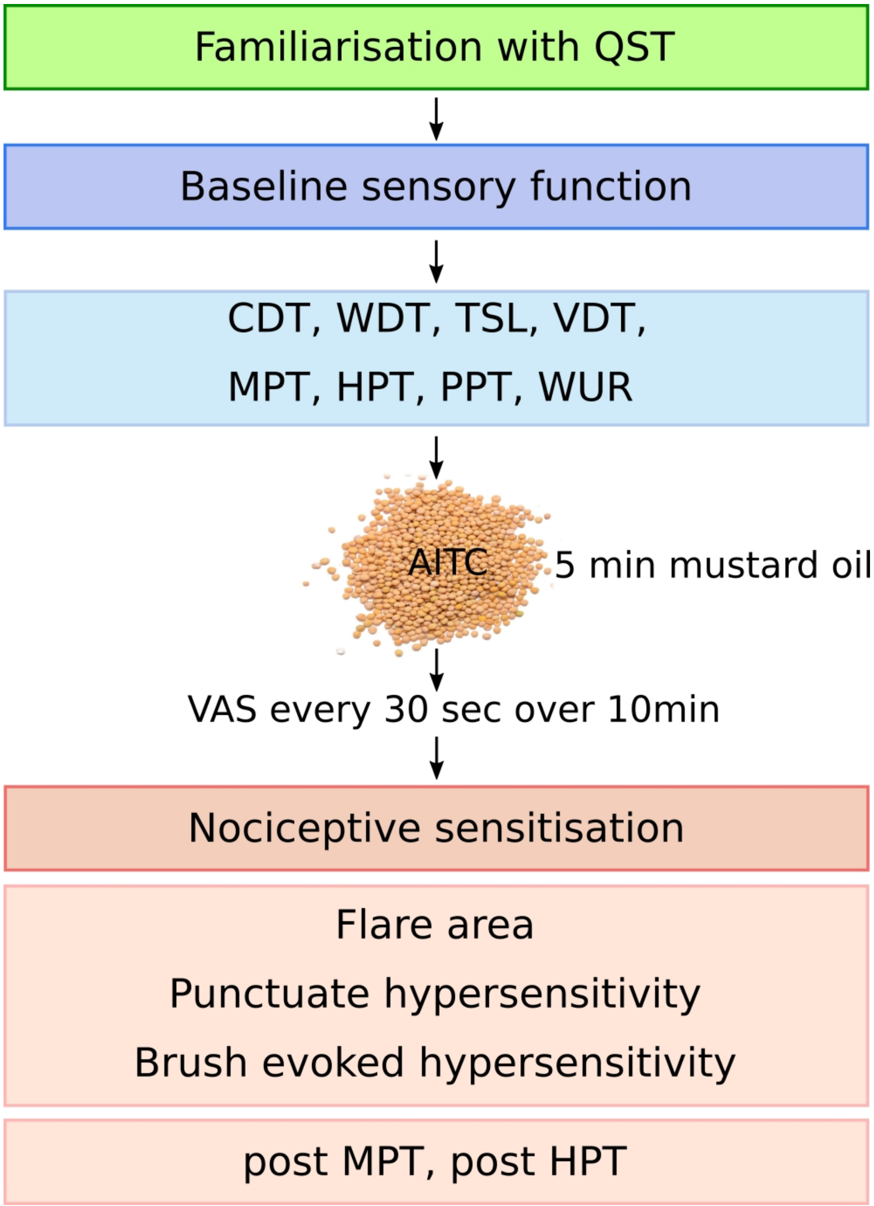

Figure 1: Study procedure. The graph demonstrates the study procedure which starts with familiarisation of the participants with QST, followed by baseline sensory function measurements. Mustard oil (AITC) will subsequently be applied for 5 minutes. After removal of mustard oil, a pain VAS will be taken every 30 seconds for 10 minutes. Subsequently, the nociceptive sensitisation measurements will be taken. CDT, WDT: cold, warm detection thresholds; TSL: thermal sensory limen; VDT: vibration detection threshold; MPT: mechanical pain threshold; HPT: heat pain threshold; PPT: pressure pain threshold; WUR: windup ratio

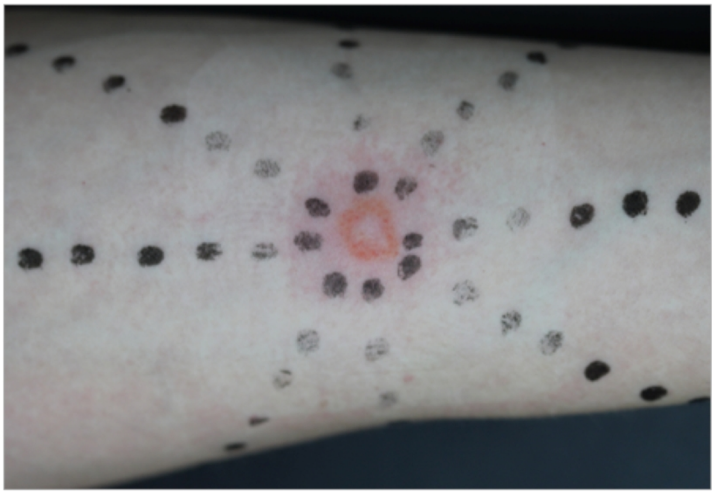

Figure 2: Method to determine area of flare, punctuate hyperalgesia and allodynia.

175x254mm (300 x 300 DPI)

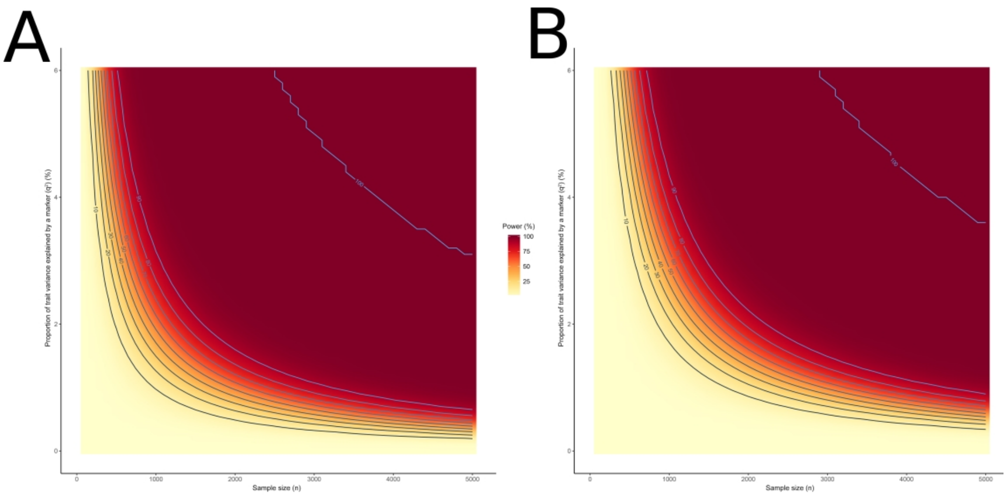

Figure 3: Estimated power. (A) estimated power (in percentage) setting the significance threshold at 5x10-8, the commonly used threshold for genome-wide significance in GWAS studies. (B) estimated power with the significance threshold set at 10-5, the commonly used threshold for suggestive significance. The X-axis denotes a range of sample sizes (n) in a GWAS, the Y-axis represents the proportion of trait variance (q2) explained by a causal marker. Power of detecting the causal marker at a specific (n, q2) combination is represented by a color gradient. Contour lines for power at 10% intervals are also shown.

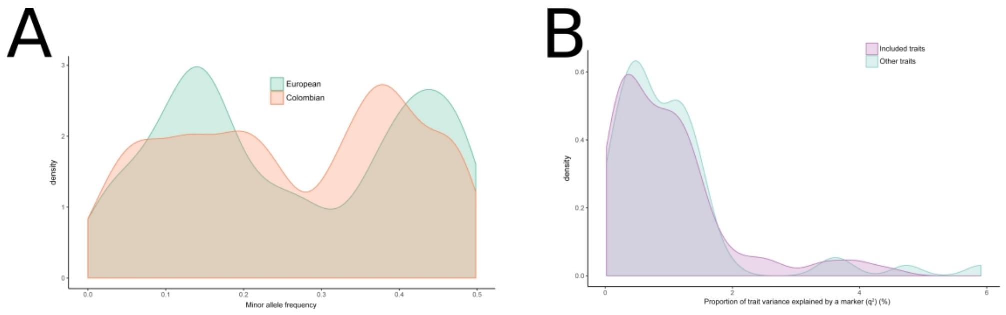

Figure 4: (A) Comparable distributions of trait variance explained by a single marker from Doehring et al35 for traits included in our study and those not included. (B) Allele frequency distributions of associated loci for Europeans and Colombians taken from previously published cohorts.

Reporting checklist for genetic association study.

Based on the STREGA guidelines.

Instructions to authors

Complete this checklist by entering the page numbers from your manuscript where readers will find each of the items listed below.

Your article may not currently address all the items on the checklist. Please modify your text to include the missing information. If you are certain that an item does not apply, please write "n/a" and provide a short explanation.

Upload your completed checklist as an extra file when you submit to a journal.

In your methods section, say that you used the STREGA reporting guidelines, and cite them as:

Little J, Higgins JP, Ioannidis JP, Moher D, Gagnon F, von Elm E, Khoury MJ, Cohen B, Davey-Smith G, Grimshaw J, Scheet P, Gwinn M, Williamson RE, Zou GY, Hutchings K, Johnson CY, Tait V, Wiens M, Golding J, van Duijn C, McLaughlin J, Paterson A, Wells G, Fortier I, Freedman M, Zecevic M, King R, Infante-Rivard C, Stewart A, Birkett N; STrengthening the REporting of Genetic Association Studies. STrengthening the REporting of Genetic Association Studies (STREGA): An Extension of the STROBE Statement.

		Reporting Item	Page Number
Title	#1a	Indicate the study's design with a commonly used term in the title or the abstract	1
Abstract	#1b	Provide in the abstract an informative and balanced summary of what was done and what was found	3
	#2	Explain the scientific background and rationale for the investigation being reported	5-7
	#3	State specific objectives, including any prespecified hypotheses. State if the study is the first report of a genetic association, a replication effort, or both.	7
	#4	Present key elements of study design early in the paper	7
	#5	Describe the setting, locations, and relevant dates, including periods of recruitment, exposure, follow-up, and data collection	7

For peer review only

#6a	Cohort study – Give the eligibility criteria, and the sources and methods of selection of participants. Describe methods of follow-up. Case-control study – Give the eligibility criteria, and the sources and methods of case ascertainment and control selection. Give the rationale for the choice of cases and controls. Cross-sectional study – Give the eligibility criteria, and the sources and methods of selection of participants. Give information on the criteria and methods for selection of subsets of participants from a larger study, when relevant.	7
#6b	Cohort study – For matched studies, give matching criteria and number of exposed and unexposed. Case-control study – For matched studies, give matching criteria and the number of controls per case.	NA
#7a	Clearly define all outcomes, exposures, predictors, potential confounders, and effect modifiers. Give diagnostic criteria, if applicable	8-10
#7b	Clearly define genetic exposures (genetic variants) using a widely-used nomenclature system. Identify variables likely to be associated with population stratification (confounding by ethnic origin).	7-10
#8a	For each variable of interest give sources of data and details of methods of assessment (measurement). Describe comparability of assessment methods if there is more than one group. Give information separately for for exposed and unexposed groups if applicable.	8-10
#8b	Describe laboratory methods, including source and storage of DNA, genotyping methods and platforms (including the allele calling algorithm used, and its version), error rates and call rates. State the laboratory / centre where genotyping was done. Describe comparability of laboratory methods if there is more than one group. Specify whether genotypes were assigned using all of the data from the study simultaneously or in smaller batches.	11
#9a	Describe any efforts to address potential sources of bias	NA
#9b	Describe any efforts to address potential sources of bias	NA
#10	Explain how the study size was arrived at	11-14
#11	Explain how quantitative variables were handled in the analyses. If applicable, describe which groupings were chosen, and why. If applicable, describe how effects of treatment were dealt with.	15-16
#12a	Describe all statistical methods, including those used to control for	15-16

For peer review only

	confounding. State software version used and options (or settings) chosen.	
#12b	Describe any methods used to examine subgroups and interactions	15-16
#12c	Explain how missing data were addressed	11
#12d	If applicable, explain how loss to follow-up was addressed	NA
#12e	Describe any sensitivity analyses	
#12f	State whether Hardy-Weinberg equilibrium was considered and, if so, how.	
#12g	Describe any methods used for inferring genotypes or haplotypes	15-16
#12h	Describe any methods used to assess or address population stratification.	NA
#12i	Describe any methods used to address multiple comparisons or to control risk of false positive findings.	15-16
#12j	Describe any methods used to address and correct for relatedness among subjects	15-116
#13a	Report numbers of individuals at each stage of study—eg numbers potentially eligible, examined for eligibility, confirmed eligible, included in the study, completing follow-up, and analysed. Give information separately for for exposed and unexposed groups if applicable. Report numbers of individuals in whom genotyping was attempted and numbers of individuals in whom genotyping was successful.	NA
#13b	Give reasons for non-participation at each stage	NA
#13c	Consider use of a flow diagram	Figure 1
#14a	Give characteristics of study participants (eg demographic, clinical, social) and information on exposures and potential confounders. Give information separately for exposed and unexposed groups if applicable. Consider giving information by genotype	NA
#14b	Indicate number of participants with missing data for each variable of interest	NA
#14c	Cohort study – Summarize follow-up time, e.g. average and total amount.	NA

For peer review only

#15	Cohort study Report numbers of outcome events or summary measures over time.Give information separately for exposed and unexposed groups if applicable. Report outcomes (phenotypes) for each genotype category over time Case-control study – Report numbers in each exposure category, or summary measures of exposure.Give information separately for cases and controls . Report numbers in each genotype category. Cross-sectional study – Report numbers of outcome events or summary measures. Give information separately for exposed and unexposed groups if applicable. Report outcomes (phenotypes) for each genotype category	NA
#16a	Give unadjusted estimates and, if applicable, confounder-adjusted estimates and their precision (eg, 95% confidence interval). Make clear which confounders were adjusted for and why they were included	NA
#16b	Report category boundaries when continuous variables were categorized	NA
#16c	If relevant, consider translating estimates of relative risk into absolute risk for a meaningful time period	NA
#16d	Report results of any adjustments for multiple comparisons	NA
#17a	Report other analyses done—e.g., analyses of subgroups and interactions, and sensitivity analyses	NA
#17b	Report other analyses done—e.g., analyses of subgroups and interactions, and sensitivity analyses	NA
#17c	Report other analyses done—e.g., analyses of subgroups and interactions, and sensitivity analyses	NA
#18	Summarise key results with reference to study objectives	NA
#19	Discuss limitations of the study, taking into account sources of potential bias or imprecision. Discuss both direction and magnitude of any potential bias.	4
#20	Give a cautious overall interpretation considering objectives, limitations, multiplicity of analyses, results from similar studies, and other relevant evidence.	16
#21	Discuss the generalisability (external validity) of the study results	NA

For peer review only

#22	Give the source of funding and the role of the funders for the present study and, if applicable, for the original study on which the present article is based	17

The STREGA checklist is distributed under the terms of the Creative Commons Attribution License CC-BY. This checklist can be completed online using https://www.goodreports.org/, a tool made by the EQUATOR Network in collaboration with Penelope.ai

For peer review only

Genetic components of human pain sensitivity: a protocol for a genome-wide association study of experimental pain in healthy volunteers

Journal:	*BMJ Open*
Manuscript ID	bmjopen-2018-025530.R1
Article Type:	Protocol
Date Submitted by the Author:	18-Jan-2019

Complete List of Authors:	Schmid, Annina B.; Oxford University, Nuffield Department of Clincial Neurosciences<br>Adhikari, Kaustubh; University College London, London, UK, Department of Genetics, Evolution and Environment; University College London, Department of Cell and Developmental Biology<br>Ramirez-Aristeguieta, Luis Miguel; Universidad de Antioquia, QST Lab. Faculty of Odontology<br>Chacón-Duque, Juan-Camilo; university College London, Department of Genetics, Evolution and Environment<br>Poletti, Giovanni; Universidad Peruana Cayetano Heredia, Unidad de Neurobiologia Molecular y Genética, Laboratorios de Investigación y Desarrollo, Facultad de Ciencias y Filosofía<br>Gallo, Carla; Universidad Peruana Cayetano Heredia, Unidad de Neurobiologia Molecular y Genética, Laboratorios de Investigación y Desarrollo, Facultad de Ciencias y Filosofía<br>Rothhammer, Francisco; Tarapacá University, Instituto de Alta Investigación<br>Bedoya, Gabriel; Universidad de Antioquia, GENMOL (Genética Molecular)<br>Ruiz-Linares, Andres; Fudan University, Ministry of Education Key Laboratory of Contemporary Anthropology and Collaborative Innovation Center of Genetics and Development, School of Life Sciences and Human Phenome Institute; Aix-Marseille Université, CNRS, EFS, ADES<br>Bennett, David; Oxford University, Nuffield Department of Clincial Neurosciences

**Primary Subject Heading**:	Genetics and genomics
Secondary Subject Heading:	Neurology, Anaesthesia
Keywords:	GENETICS, nociception, pain sensitivity, pain, genome wide association study, hyperalgesia

SCHOLARONE™
Manuscripts

Genetic components of human pain sensitivity: a protocol for a genome-wide association study of experimental pain in healthy volunteers

Annina B Schmid[1*], Kaustubh Adhikari[2,3*], Luis-Miguel Ramirez-Aristeguieta[4], Juan-Camilo Chacón-Duque[2], Giovanni Poletti[5], Carla Gallo[5], Francisco Rothhammer[6], Gabriel Bedoya[7], Andres Ruiz-Linares[8,9], David L Bennett[1]

1 Nuffield Department of Clinical Neurosciences, Oxford University, Oxford, UK

2 Department of Genetics, Evolution and Environment, University College London, London, UK

3 Department of Cell and Developmental Biology, University College London, London, UK

4 QST Lab. Faculty of Odontology, University of Antioquia, Medellin, Colombia

5 Unidad de Neurobiologia Molecular y Genética, Laboratorios de Investigación y Desarrollo, Facultad de Ciencias y Filosofía, Universidad Peruana Cayetano Heredia, Lima, Peru

6 Instituto de Alta Investigación, Tarapacá University, Chile;

7 GENMOL (Genética Molecular), Universidad de Antioquia, Medellín, Colombia

8 Ministry of Education Key Laboratory of Contemporary Anthropology and Collaborative Innovation Center of Genetics and Development, School of Life Sciences and Human Phenome Institute, Fudan University, Shanghai, China.

9 Aix-Marseille Université, CNRS, EFS, ADES, Marseille, France

* authors contributed equally to this work.

Corresponding authors:

David LH Bennett

For peer review only

Oxford University, Nuffield Department of Clinical Neurosciences

John Radcliffe Hospital

West Wing Level 6

OX39DU Oxford

United Kingdom

Phone: +44 (0)1865 231512

Andrés Ruiz-Linares

Ministry of Education Key Laboratory of Contemporary Anthropology and Collaborative

Innovation Center of Genetics and Development

Fudan University

2005#Songhu Road, Yangpu District

Shanghai 200438, China

86-21-51630503

Corresponding authors email addresses:

David.bennett@ndcn.ox.ac.uk

andresruiz@fudan.edu.cn

Word count: 3786

Keywords: genetics, pain, nociception, pain sensitivity, hyperalgesia, genome-wide association study

For peer review only

Abstract:

Introduction: Pain constitutes a major component of the global burden of diseases. Recent studies suggest a strong genetic contribution to pain susceptibility and severity. Whereas most of the available evidence relies on candidate gene association or linkage studies, research on the genetic basis of pain sensitivity using genome-wide association studies (GWAS) is still in its infancy. This protocol describes a proposed GWAS on genetic contributions to baseline pain sensitivity and nociceptive sensitization in a sample of unrelated healthy individuals of mixed Latin American ancestry.

Methods and Analysis: A genome-wide association study (GWAS) on genetic contributions to pain sensitivity in the naïve state and following nociceptive sensitization will be conducted in unrelated healthy individuals of mixed ancestry. Mechanical and thermal pain sensitivity will be evaluated with a battery of quantitative sensory tests (QST) evaluating pain thresholds. In addition, variation in mechanical and thermal sensitisation following topical application of mustard oil to the skin will be evaluated.

Ethics and Dissemination: This study received ethical approval from the University College London research ethics committee (3352/001) and from the bioethics committee of the Odontology Faculty at the University of Antioquia (CONCEPTO 01-2013). Findings will be disseminated to commissioners, clinicians and service users via papers and presentations at international conferences.

For peer review only

Article Summary

Strengths and Limitations of this study

- We propose a genome-wide association study (GWAS) of both baseline pain sensitivity as well as nociceptive sensitisation.
- The study will be conducted in an admixed population in Colombia, with variable proportions of European, Native American and Sub-Saharan African ancestry.
- Phenotypic data will be collected by a single trained examiner, thus producing high quality measures.
- Because we are focusing on highly reproducible pain phenotypes obtained at a single centre, our initial sample size is limited to 1500-2000 participants. This will allow us to identify only genetic variants that have intermediate and large (but not small) effect sizes.

For peer review only

INTRODUCTION

Pain constitutes a major component of the global burden of diseases, with lower back and neck pain representing the single leading cause for years lived with disability followed closely by migraine and other musculoskeletal disorders[1]. Pain is a multidimensional experience involving a highly complex interaction of physical, biochemical, physiological, cognitive, emotional, behavioural and sociocultural factors. Many studies have identified genetic factors in a range of chronic pain conditions[2]. Importantly, a growing number of studies in patient populations suggest that genetics is an important contributory factor to pain susceptibility and severity[2-4]. Of note, twin studies using either clinical pain outcomes or experimental pain models suggest that sensitivity to pain has a heritability of up to 55%[5-10]. Interestingly, the heritability varies greatly depending on the clinical pain outcome or the sensory modality tested in experimental pain models[7]. There is evidence that different sensory modalities may have distinct genetic components contributing to their variance in humans,[10] and this is consistent with animal models which underline the distinct neurobiology mediating different sensory modalities[11].

A powerful technology for identifying genetic determinants of human complex phenotypes are genome-wide association studies (GWAS). However, GWAS analyses of pain remain limited, mainly due to the high numbers of individuals required to enable adequate power, and the complexities of accurately phenotyping traits that ultimately represent a personal subjective perception[2]. In contrast to disease cohorts where pain variation may be influenced by the severity of the disease process or its treatment, experimental pain studies measuring baseline pain sensitivity have the advantage of studying one stimulus in a standard condition (e.g., controlling intensity, location and stimulus duration). To date, several candidate gene studies have been performed to determine genetic influences on human pain sensitivity using

For peer review only - http://bmjopen.bmj.com/site/about/guidelines.xhtml

experimental pain models[5-9 12 13], however, to our knowledge, a full GWAS has not yet been reported. One genome-wide study evaluated association for SNPs revealed by exome sequencing in a sub-set of a twins cohort that were identified as having particularly high or low heat pain sensitivity. Using pathway analysis there was significant enrichment for variants in genes of the angiotensin pathway[14]. Whereas a direct link between experimental pain sensitivity and clinical pain severity is often not present[15], there is some evidence that findings from experimental pain models can be predictive of clinically relevant pathological pain such as post-operative pain[16]. Irrespective of the association between experimental and pathological pain, understanding the genetic influences on experimental pain sensitivity will provide important biological insights into the mechanisms underlying pain sensitivity.

An important drawback of most genetic studies in the pain field so far is that they have been performed mostly in populations of European ancestry, thus they have explored only a small fraction of human phenotypic and genetic diversity[17 18]. This is important in the study of pain as recent studies point to variable pain thresholds for European Americans, African Americans and Latinos[19-21]. However, it is not clear if this variation in pain thresholds relates to differences in neurobiological mechanisms or other factors such as social or cultural[22] parameters. In addition, recent studies suggest that a phenotype associated with increased sensitization of the nociceptive system due to temporal summation may render people vulnerable to developing clinically relevant pain[23]. However, most studies focus on pain phenotyping in the naïve state rather than sensitized state; only a handful of studies have investigated a genetic component underlying nociceptive sensitization[7 24]. We therefore intend to use an algogen (AITC: Allyl isothiocyanate, an agonist of the ligand gated ion channel TRPA1) in order to sensitise the nociceptive system to replicate the changes that could occur in pathological pain states.

For peer review only - http://bmjopen.bmj.com/site/about/guidelines.xhtml

This protocol proposes a GWAS on genetic contributions to baseline pain sensitivity and nociceptive sensitization in a sample of unrelated healthy individuals of mixed European/Native American/African ancestry. We will evaluate baseline cutaneous pain thresholds as well as the variation in sensitisation following mustard oil (AITC) application, a controlled model of tissue injury. We hypothesise that we will identify single nucleotide polymorphisms (SNPs) associated with experimental pain stimuli in the naïve and sensitized state. Elucidating the genetic basis of pain variation has the potential to reveal targets for future analgesic development. This can be translated into improved pain management potentially tailored to an individual's pain risk or resilience, including sensitivity differences between different human populations.

METHODS AND ANALYSIS:

This GWAS follows the strengthening the reporting of genetic association studies guidelines (STREGA)[25]. A flowchart of the study procedure is detailed in Figure 1.

Participants

Healthy participants aged 18-40 will be recruited in Medellin, Colombia via public noticeboards at local Universities, distribution of flyers and through the local print media. In addition, we are inviting previous participants from the CANDELA (Consortium for the Analysis of the Diversity and Evolution of Latin America)[26] GWAS to participate in this project.

Recruiting healthy young participants has advantages in GWAS studies. They are less likely to have undetected illnesses or other problems that may influence their biological pathway of pain sensitivity. Young people will also have less overall accumulated exposure or risks from

For peer review only

environmental (external) factors which may affect their pain sensitivity. Such factors increase the overall variability of participants' pain perception response and reduce the power of detecting genetic causes. Since most traits are affected by a combination of genetic and environmental factors, many studies including CANDELA tend to use young participants for genetic variant discovery[27].

Participants will be excluded if they have chronic pain or any chronic medical condition (e.g, diabetes, neurodegenerative, musculoskeletal or psychiatric conditions). Participants currently taking analgesics, anti-inflammatories, opioids, antihistamines, antidepressants, or anti-epilectic drugs will be excluded. Women who are pregnant or in their menstrual phase (self-report) will be excluded from the study. Participants will be advised to not smoke or consume coffee within 1 hour of testing, and to avoid psycho-active substances or alcohol within 8 hours prior to testing. Further exclusion criteria include current or past self-inflicted injuries, as well as dermatomal, traumatic, or infectious conditions affecting the arm, and a history of severe allergic reactions to any kind of medication, materials, food, or insect bites. Participants with moderate to severe anxiety (≥25 on the Hamilton Anxiety Rating Scale[28]) or severe depression (>15 on the Quick Inventory of Depressive Symptomatology (QIDS-SR16)[29]) will be excluded from the study. Recruitment started in January 2013 and is predicted to take approximately 5-7 years.

Procedure

Participants will attend a single appointment at the quantitative sensory testing (QST) laboratory at the Universidad de Antioquia, Medellín. Following informed consent, age and self-reported gender will be recorded and participants will answer questions regarding their self-reported ancestry (see Appendix 1). Height and weight will be measured and BMI (body

For peer review only

mass index) calculated. Since psychological factors such as anxiety can influence pain perception during experimental pain testing[30], participants will complete the Spanish version of the Hamilton Anxiety Rating Scale and the QIDS-SR16. The QIDS-SR16 has acceptable internal consistency and moderate to strong concurrent validity compared to other depression scores[31] and its Spanish version shows adequate test-retest reliability and high internal consistency[32]. The Hamilton Anxiety Rating scale has shown to have high inter-rater and test-retest reliability[33] and good construct validity[34].

For peer review only

Evaluation of sensory function in the naïve state

We will determine sensory function in the naïve state and following nociceptive sensitization. Baseline sensory function will be evaluated using specific static and dynamic quantitative sensory tests. These include cold and warm detection thresholds (CDT, WDT), thermal sensory limen (TSL) and heat pain thresholds (HPT) using a thermotester (Q-sense, Medoc, Israel, 30x30mm thermode size). Recording of thermal thresholds will strictly follow published quantitative sensory testing guidelines[35]. Mechanical pain thresholds will be evaluated using a 20 piece von Frey hair set (Touch Test, North Coast, USA) wich exerts differening forces (9.8, 13.7, 19.6, 39.2, 58.8, 78.5, 98.1, 147.1, 255.0, 588.4, 980.7, 1765.2, 2942.0 mN). The von Frey hairs will be applied at a rate of 2s on, 2s off in ascending order starting from 9.8mN baseline stimulus until participants first perceive the stimulus as sharp (pricking). Subsequently, the hairs will be applied in descending order until the stimulus is perceived as blunt. The geometric mean of five series of ascending and descending stimuli is defined as the mechanical pain threshold (MPT). Wind-up ratio (WUR) will be determined with numerical pain ratings in a visual analogue scale (VAS 0-100) for a single stimulus followed by the average pain rating for a train of 10 stimuli applied at 1 Hz within the same $1cm^2$ using a 255mN von Frey hair. This will be repeated five times and the ratio will be

For peer review only - http://bmjopen.bmj.com/site/about/guidelines.xhtml

established as the mean rating of the trains of stimuli divided by the mean rating of the single stimuli. Vibration detection thresholds (VDT) will be determined by recording the mean of 3 disappearance thresholds with a Rydel-Seiffer tuning fork. Pressure pain thresholds (PPT) will be recorded in triplicate with a manual algometer (Wagner Instruments, USA) and their mean used for analysis.

The side to be tested will be randomized and patients will first be familiarized with the sensory tests on the forearm on the control side, before performing the actual measurements on the test arm. All tests will be performed half way over the volar side of the forearm except for VDT (ulnar styloid) and PPT (thenar muscles).

Mustard oil evoked nociceptive sensitisation

After the baseline sensory measures, an acetate template will be used to mark a star with eight spokes each containing eight points at 1cm increments on the volar forearm (Figure 2). The skin temperature will be standardised by placing the 32° C warm thermode on the volar forearm for 5 minutes before starting. We will then apply a sensitization paradigm using mustard oil (AITC: Allyl isothiocyanate (Sigma), diluted at 30% in olive oil) as previously performed[36]. AITC, the active component of mustard oil, activates the ion channel TRPA1 and evokes skin flare and nociceptive sensitisation[37]. A small cotton swab soaked in mustard oil will be applied to a 0.64 cm^2 area on the volar forearm and held in place with a tegaderm (3M) for 10 minutes[36]. During this time, pain scores will be recorded every 30 seconds using an electronic visual analogue scale (VAS) ranging from 0 to 100. After 10 minutes, the mustard oil will be removed and the area of the skin flare will be recorded to the nearest 0.5 cm at each spoke[7]. Eight triangular shapes will be created by joining the points on adjacent spokes and the total area will be calculated by adding all triangular segments. The area of

For peer review only - http://bmjopen.bmj.com/site/about/guidelines.xhtml

mustard oil application will be subtracted from the total area to determine the area of secondary flare (flare area).

After mapping of the flare, the area of brush-evoked hypersensitivity will be determined with a brush (Nr 5 Senselab, Somedic, Sweden) by applying 1 cm long strokes at each of the points on the eight spokes, starting from the outside and moving towards the sensitized centre. The area of punctuate hypersensitivity will be determined with a 98.1mN filament (Bailey Instruments, UK) following the same procedure[36]. As for the flare, the primary area of mustard oil application will be subtracted from both hypersensitive areas such that the recorded areas represent secondary hyperalgesia/hypersensitivity.

Following mustard oil sensitization, the mechanical and heat pain thresholds (MPT, HPT) will be repeated using the same methods as described above. All post-sensitisation tests will be performed within 5 minutes of mustard oil removal.

Reliability of naïve and sensitized sensory function protocol

To determine intratester reliability, we repeated the sensory function protocol performed by the same investigator in n=12 healthy volunteers on two different occasions within 2-6 weeks. Intraclass correlation coefficients (ICC 3.1) revealed good to excellent agreement for all sensory testing variables (Table 1).

Genotyping

Each participant will donate blood or saliva (Oragene OG-500, Genotek, Canada) for DNA extraction. DNA samples will be genotyped on the Illumina HumanOmniExpress chip containing ~700,000 markers. In volunteers who already participated in the CANDELA

For peer review only - http://bmjopen.bmj.com/site/about/guidelines.xhtml

GWAS[38], genotype data from blood samples genotyped on the same chip is already available and will be reused.

Whole-genome genotype data from the Illumina array will undergo quality control[39] to exclude any markers or samples that fail stringent thresholds. Quality metrics provided by the genotype calling algorithm in the Illumina GenomeStudio software[40], such as the GenTrain score, cluster separation score, and excess heterozygosity rates will be used to filter poorly genotyped SNPs. Subsequent SNP- and sample-level quality control thresholds such as missingness will be applied. Sex mismatch between records and genetic data of X & Y chromosomes will be checked. Only samples and SNPs that pass all criteria will be retained for analysis. Details of the currently used quality control protocol for CANDELA genotyped samples are provided in Appendix 2.

Statistical analysis

Sample size calculation

The power for Genome-wide association studies of experimental pain phenotypes for varying sample and effect sizes was estimated following the formulae described in Visscher et al[41]. Estimated power is shown for a range of effect sizes for experimental pain phenotypes taken from existing experimental pain studies. The statistical software R version 3.4.1[42] was used to perform the calculations and produce the figures. The codes are published on https://github.com/kaustubhad/gwas-power.

In whole-genome SNP-based GWAS studies, the association analysis is usually conducted with a multivariate linear regression model, where the trait values are regressed onto a SNP genotype (with additive coding) and other covariates which commonly include age, gender,

For peer review only

For peer review only - http://bmjopen.bmj.com/site/about/guidelines.xhtml

BMI, and genetic principal components (PCs). The p-value threshold [38 41] for genome-wide significant associations is commonly 5×10^{-8}, while the threshold for a suggestive significant association is commonly 10^{-5}. Formulae to calculate power in GWAS with genome-wide and suggestive significance thresholds are presented in Appendix 3, and power calculated for the current GWAS setting is shown in Figure 3.

Figure 3A shows estimated power (in percentage) as a heatmap under the standard GWAS settings of using whole-genome genotyping data and a p-value significance threshold of 5×10^{-8}. Sample size (n) varies from 100 to 5000, while the proportion of trait variance explained by the marker (q^2, in percentage) varies from 0.01% to 6%. As sample size increases, power increases quickly for a range of trait variance values to reach 100%.

Figure 3B shows estimated power (in percentage) under the same settings but a suggestive p-value significance threshold of 10^{-5}. As expected, power is higher at similar sample and effect sizes for this less stringent threshold.

Simplified power estimates are shown as power curves in Figure 3C-D for the expected sample sizes for this study. Figure 3C shows expected power at genome-wide and suggestive significance thresholds for a sample size of n=1500 at varying effect sizes, while Figure 3D shows estimated power for a sample size of n=2000.

The range of trait variance has been taken from Doehring et al[43], which provides estimates for the proportion of trait variance explained by a SNP for several experimental pain phenotypes and multiple markers. The values ranged from 0.02% to 6%. Some of the traits were the same as the traits investigated here, while some other traits were different.

For peer review only - http://bmjopen.bmj.com/site/about/guidelines.xhtml

Nevertheless, the distributions of trait variance for the two groups of traits are very similar, as seen in Figure 4A.

Power of a GWAS depends on the allele frequency of the SNPs through their effect on the test statistic. While the majority of GWAS studies are conducted in European-origin individuals, including the experimental pain study used to determine sample size here[43], our population of interest is an admixed Latin American population. Therefore, we wanted to assess the distribution of allele frequencies in Europeans versus Colombians for SNPs studied for or associated with experimental pain in various studies[43 44]. Minor allele frequencies were obtained for all such reported SNPs from the 1000 Genomes project database[17] for Western Europeans (from Britain (GBR), Utah residents from Northern & Western Europe (CEU), Spain (IBS), and Tuscany in Italy (TSI)) and Colombians (from Medellin in Colombia, (CLM), where this study will be performed). Allele frequency distributions for both Europeans and Colombians are shown in Figure 4B. The two distributions are quite similar, with the Colombian distribution slightly more spread out. This is somewhat expected as the Colombians have on average 60% European (Spanish) ancestry[26]. Having a well spread out distribution of allele frequencies is important in a GWAS as low-frequency alleles have lower power for a given sample and effect size[45]. Here, the comparison to European allele frequency distribution suggests that the current Colombian cohort will have nearly equivalent power to any European-based cohort. In contrast to European-only cohorts though, our cohort will have the advantage that alleles present in other continental populations such as Sub-Saharan Africans or Native Americans that are not present in Europeans would also be detectable in a GWAS, and could be followed up in replication cohorts of specific ethnicities.

For peer review only - http://bmjopen.bmj.com/site/about/guidelines.xhtml

The CANDELA project includes genotpyes of ~2000 patients from Medellin. We anticipate to contact and phenotype 50-75% of these participants as well as contacting an additional 500 participants to bring the initial sample to 1500-2000.

Data analysis plan

The cleaned genetic data will first be merged with reference samples worldwide, such as the 1000 Genomes Project[17], Simons Genome Diversity Project[46], Estonian Biocentre Human Genome Diversity Panel[47], and additional European and Native American samples that are particularly relevant for Latin American populations[48]. The merged dataset will be checked for genetic outliers, through genetic principal components (PCs) and continental ancestry proportions (using supervised Admixture[49]), and for unexpected genetic similarities. These steps can often detect any sample misplacement or contamination, which might be reflected in sex mismatch, unexpected genetic similarities, or inflated heterozygosity rates[39 50]. Genetic ancestry estimates will be compared to self-reported ancestry information (see Appendix 1), particularly for genetic outliers or samples showing unexpected results. Participants self-reporting for ethnicities rare in Colombians, such as East Asian or South Asian, would also be excluded as outliers. The authors have extensive experience in conducting association analysis in admixed populations, including several GWAS publications on a wide range of phenotypes which contain detailed protocols on how to conduct such analyses[51 27 38]. Further details of the currently used quality control protocol for CANDELA samples[51] are provided in Appendix 2.

The genetic data allows estimation of the narrow-sense heritability of any quantitative trait, which is the fraction of trait variance that is explained by the genetic data. Estimates of heritability, obtained using the software GCTA[52] will provide an idea of which traits have more of a biological basis versus which are more environmentally determined, and thus

For peer review only - http://bmjopen.bmj.com/site/about/guidelines.xhtml

which traits would be more amenable to genetic analysis for discovery of associated genetic variants. Note however that relatively precise estimates (low standard errors) of heritability by this method requires several thousand samples[51], so the currently proposed sample size might be under-powered to estimate heritability accurately.

To facilitate better identification of associated loci, the genotype data will be imputed to approximately 10 million loci using the 1000 Genomes Phase 3 imputation reference panel[51] by first haplotype phasing using SHAPEIT2[53] and then imputation using Impute2[54]. Quality control of the imputed genotypes will be performed using recommended thresholds on imputation quality score, concordance metrics, and proportion of high-probability calls. Details of the currently used imputation protocol for CANDELA samples[51] are provided in Appendix 2.

GWAS studies will be conducted in Plink2[55] to perform single-locus association studies for each trait individually, across the whole genome in an additive multivariate linear regression model[41]. Covariates will be used in the regression to adjust for any other sources of trait variability, such as basic variables like age, sex, and BMI, and genetic principal components will be used to control for population substructure[51 56].

The number of genetic PCs to be included in the regression depends on the sample composition, such as variation in ancestry and presence/absence of genetic outliers. It would be determined by inspecting the proportion of variance explained by each PC (displayed on a scree plot) and by checking PC scatter plots.

In addition to being used as exclusion criteria, anxiety and depression scores could be used as covariates in GWAS. The exact set of covariates to be used will be determined based on initial diagnostic analyses such as correlation analysis.

For peer review only - http://bmjopen.bmj.com/site/about/guidelines.xhtml

These single-locus association results, obtained as P-values, will be visualized via the Manhattan plot. Commonly used P-value thresholds for selecting associated loci are 5×10^{-8} for genome-wide significance and 10^{-5} for suggestive significance[41].

An extension of this additive multivariate linear regression model, still within the single-trait single-locus setting, called the mixed linear mode analysis (MLMA) which better controls for any cryptic relatedness or population substructure, will also be performed in GCTA[52]. There are several extensions of the single-trait single-locus association studies that increase power for detecting associated loci: combining several related traits that may share a biological basis, using multivariate Wald tests as implemented in MultiPhen[57]; or gene-based tests that combine signals across all loci in a gene to increase signal strength and reduce the burden of multiple testing, such as set-based models implemented in Plink2[55] or fastBAT implemented in GCTA[58]. The admixed nature of the sample might be utilized in detecting associations by the method of admixture mapping[59], though the potential of success of this method in detecting associated variants depends on the extent of stratification of the variant's allele frequency across continents. These analyses might help detect additional loci that are under-powered in classical GWAS due to smaller effect sizes.

Handling of missing data

It might not be possible to record some traits in some individuals, even though the completeness of the first 100 samples suggests that missingness will be low. The single-trait methods used in traditional GWAS analyses automatically exclude individuals from the analysis of a trait who have missing values for that trait. The same applies to individuals having missing genotypes for any particular SNP. However, genotyping success rates using

For peer review only - http://bmjopen.bmj.com/site/about/guidelines.xhtml

the Illumina HumanOmniExpress chip in the CANDELA cohort is very high (>99.8%), so the number of excluded individuals in any analysis would be very low overall.

Some multivariate analyses such as principal components when applied on the set of phenotypes require having recorded values of all phenotypes for an individual. Instead of using the subset of individuals who have the complete set of phenotypes recorded, which would incur some loss in sample size, the missing phenotype data for each individual will be imputed following standard statistical procedures as implemented in the R package 'mice'[60]. When the proportion of missing data is small, imputation is preferable in such multivariate analyses than sample exclusion, and is routinely applied to genetic data such as while calculating genetic PCs[56].

DISCUSSION:

This GWAS including a well-defined cohort of healthy participants will provide important insights into the genetic aspects underlying experimental pain sensitivity in the naïve and sensitized state. This may allow further exploration of potential biological mechanisms underlying pain sensitivity. Future studies will be required to extrapolate these findings to patient populations with chronic pain.

Patient and Public Involvement

No patient involvement is performed during this study.

For peer review only - http://bmjopen.bmj.com/site/about/guidelines.xhtml

Ethics and dissemination:

This study received ethical approval from the University College London research ethics committee (3352/001) and from the bioethics committee of the Odontology Faculty at the University of Antioquia (CONCEPTO 01-2013). Findings will be disseminated to commissioners, clinicians and service users via papers and presentations at international conferences such as the biennial World Congress of International Association for the Study of Pain. We will also post our findings to the publicly available painnetworks.org database.

Acknowledgements:

We thank Peter Kamerman for his input to parts of the study design, and Macarena Fuentes-Guajardo for helping with the phenotyping photographs in Figure 2.

We would like to thank the CANDELA team for their contributions in organisation, recruitment, data collection, sample processing and genotyping: Alvaro Alvarado, Ana Carolina Orozco, Caio C. Silva de Cerqueira, Claudia Jaramillo, David Balding, Diana Rogel Diaz, Francisco de Ávila Becerril, Francisco M. Salzano, Francisco Quispealaya, Hugo Villamil-Ramírez, Ilich Jafet Moreno, Javier Mendoza-Revilla, Javier Rosique, Jorge Gómez-Valdés, Joyce De la Piedra, Lavinia Schuler-Faccini, Mónica Ballesteros Romero, Major Eugênio Correa de Souza Junior, Malena Hurtado, María Pizarro, María Teresa Del Solar, Marcelo Zagonel de Oliveira, Mari-Wyn Burley, Maria-Cátira Bortolini, Martha Granados Riveros, Miguel Ángel Contreras Sieck, Paola Everardo, Paola Everardo Martínez, Paola León-Mimila, Ricardo Cebrecos, Ricardo Gunski, Rodrigo Barquera Lozano, Rolando Gonzalez-José, Rosilene Paim, Ruth Rojas, Samuel Canizales-Quinteros, Sergeant João Felisberto Menezes Cavalheiro, Tábita Hunemeier, Valeria Villegas, Vanessa Granja, Vanessa Sarabia, Victor Acuña-Alonzo, Virginia Ramallo, Wendy Hart, William Arias, and William Flores.

For peer review only - http://bmjopen.bmj.com/site/about/guidelines.xhtml

For peer review only

Author Contributions:

DLB and ARL conceptualised the study. Funding was acquired by DLB, ABS, GB and ARL. ABS, JCCD, KA, and LMR have contributed to the study methodology. ARL, KA, JCCD, GB, FR, CG and GP contributed to the design and execution of the CANDELA aspect of this study. The first draft of the protocol was prepared by ABS, DLB, KA and ARL and all authors provided critical evaluation and approved the final manuscript.

Data Availability:

Individual-level data cannot be made available due to restrictions imposed by the ethics approval. Summary statistics from the GWAS will be deposited in GWAS central at https://www. gwascentral.org/

Code Availability:

The R codes for power and sample size calculations are available at https://github.com/kaustubhad/gwas-power (see methods).

Funding statement:

Work leading to this publication was funded by grants from: the Newton Fund Institutional Links Grant from the British Research Council (grant number 216398412), the Excellence Initiative of Aix-Marseille University - A*MIDEX (a French “Investissements d’Avenir” programme), and from a Wellcome Trust Senior Scientist Fellowship to DLB (ref. no. 095698z/11/z and 202747/Z/16/Z). KA was supported by a Wellcome Investigator Award WT107055AIA (to Prof. C.D. Stern). ABS was supported by the National Institute for Health Research (NIHR) Biomedical Research Centre (BRC) Oxford. The views expressed are those of the author(s) and not necessarily those of the NHS, the NIHR or the Department of Health.

For peer review only - http://bmjopen.bmj.com/site/about/guidelines.xhtml

Competing interests statement

The authors declare no competing interest.

For peer review only

For peer review only - http://bmjopen.bmj.com/site/about/guidelines.xhtml

REFERENCES:

1. Disease GBD, Injury I, Prevalence C. Global, regional, and national incidence, prevalence, and years lived with disability for 310 diseases and injuries, 1990-2015: a systematic analysis for the Global Burden of Disease Study 2015. *Lancet* 2016;388(10053):1545-602. doi: 10.1016/S0140-6736(16)31678-6

2. Zorina-Lichtenwalter K, Meloto CB, Khoury S, et al. Genetic predictors of human chronic pain conditions. *Neuroscience* 2016;338:36-62. doi: 10.1016/j.neuroscience.2016.04.041 [published Online First: 2016/10/27]

3. Bennett DL, Woods CG. Painful and painless channelopathies. *Lancet Neurol* 2014;13(6):587-99. doi: 10.1016/S1474-4422(14)70024-9

4. Denk F, McMahon SB. Neurobiological basis for pain vulnerability: why me? *Pain* 2017;158 Suppl 1:S108-S14. doi: 10.1097/j.pain.0000000000000858

5. Belfer I, Diatchenko L. Pain Genetics: basic to translational science. Oxford: Wiley 2014.

6. Nielsen CS, Stubhaug A, Price DD, et al. Individual differences in pain sensitivity: genetic and environmental contributions. *Pain* 2008;136(1-2):21-9. doi: 10.1016/j.pain.2007.06.008 [published Online First: 2007/08/19]

7. Norbury TA, MacGregor AJ, Urwin J, et al. Heritability of responses to painful stimuli in women: a classical twin study. *Brain* 2007;130(Pt 11):3041-9. doi: 10.1093/brain/awm233

8. Hocking LJ, Generation S, Morris AD, et al. Heritability of chronic pain in 2195 extended families. *European journal of pain* 2012;16(7):1053-63. doi: 10.1002/j.1532-2149.2011.00095.x [published Online First: 2012/02/18]

For peer review only

9. Angst MS, Phillips NG, Drover DR, et al. Pain sensitivity and opioid analgesia: a pharmacogenomic twin study. *Pain* 2012;153(7):1397-409. doi: 10.1016/j.pain.2012.02.022 [published Online First: 2012/03/27]

10. Nielsen CS, Knudsen GP, Steingrimsdottir OA. Twin studies of pain. *Clin Genet* 2012;82(4):331-40. doi: 10.1111/j.1399-0004.2012.01938.x

11. Mogil JS. Pain genetics: past, present and future. *Trends Genet* 2012;28(6):258-66. doi: 10.1016/j.tig.2012.02.004

12. Kim H, Mittal DP, Iadarola MJ, et al. Genetic predictors for acute experimental cold and heat pain sensitivity in humans. *J Med Genet* 2006;43(8):e40. doi: 10.1136/jmg.2005.036079

13. Kim H, Neubert JK, San Miguel A, et al. Genetic influence on variability in human acute experimental pain sensitivity associated with gender, ethnicity and psychological temperament. *Pain* 2004;109(3):488-96. doi: 10.1016/j.pain.2004.02.027

14. Williams FM, Scollen S, Cao D, et al. Genes contributing to pain sensitivity in the normal population: an exome sequencing study. *PLoS Genet* 2012;8(12):e1003095. doi: 10.1371/journal.pgen.1003095

15. Hubscher M, Moloney N, Leaver A, et al. Relationship between quantitative sensory testing and pain or disability in people with spinal pain-a systematic review and meta-analysis. *Pain* 2013;154(9):1497-504. doi: 10.1016/j.pain.2013.05.031 [published Online First: 2013/05/29]

16. Werner MU, Mjobo HN, Nielsen PR, et al. Prediction of postoperative pain: a systematic review of predictive experimental pain studies. *Anesthesiology* 2010;112(6):1494-502. doi: 10.1097/ALN.0b013e3181dcd5a0

For peer review only - http://bmjopen.bmj.com/site/about/guidelines.xhtml

17. Genomes Project C, Auton A, Brooks LD, et al. A global reference for human genetic variation. *Nature* 2015;526(7571):68-74. doi: 10.1038/nature15393

18. Popejoy AB, Fullerton SM. Genomics is failing on diversity. *Nature* 2016;538(7624):161-64. doi: 10.1038/538161a

19. Rahim-Williams FB, Riley JL, 3rd, Herrera D, et al. Ethnic identity predicts experimental pain sensitivity in African Americans and Hispanics. *Pain* 2007;129(1-2):177-84. doi: 10.1016/j.pain.2006.12.016 [published Online First: 2007/02/14]

20. Rahim-Williams B, Riley JL, 3rd, Williams AK, et al. A quantitative review of ethnic group differences in experimental pain response: do biology, psychology, and culture matter? *Pain Med* 2012;13(4):522-40. doi: 10.1111/j.1526-4637.2012.01336.x

21. Bagwath Persad LA, Kamerman PR, Wadley AL. Predictors of Cold and Pressure Pain Tolerance in Healthy South African Adults. *Pain Med* 2017;18(11):2126-37. doi: 10.1093/pm/pnw291

22. Zborowski M. Cultural components in responses to pain. *Journal of Social Issues* 1952;8(4):16-30.

23. Weissman-Fogel I, Granovsky Y, Crispel Y, et al. Enhanced presurgical pain temporal summation response predicts post-thoracotomy pain intensity during the acute postoperative phase. *J Pain* 2009;10(6):628-36. doi: 10.1016/j.jpain.2008.12.009

24. Tegeder I, Adolph J, Schmidt H, et al. Reduced hyperalgesia in homozygous carriers of a GTP cyclohydrolase 1 haplotype. *Eur J Pain* 2008;12(8):1069-77. doi: 10.1016/j.ejpain.2008.02.004

For peer review only - http://bmjopen.bmj.com/site/about/guidelines.xhtml

25. Little J, Higgins JP, Ioannidis JP, et al. STrengthening the REporting of Genetic Association studies (STREGA)--an extension of the STROBE statement. *Eur J Clin Invest* 2009;39(4):247-66. [published Online First: 2009/03/21]

26. Ruiz-Linares A, Adhikari K, Acuna-Alonzo V, et al. Admixture in Latin America: geographic structure, phenotypic diversity and self-perception of ancestry based on 7,342 individuals. *PLoS Genet* 2014;10(9):e1004572. doi: 10.1371/journal.pgen.1004572

27. Adhikari K, Fontanil T, Cal S, et al. A genome-wide association scan in admixed Latin Americans identifies loci influencing facial and scalp hair features. *Nature communications* 2016;7:10815. doi: 10.1038/ncomms10815 [published Online First: 2016/03/02]

28. Hamilton M. The assessment of anxiety states by rating. *Br J Med Psychol* 1959;32(1):50-5.

29. Rush AJ, Trivedi MH, Ibrahim HM, et al. The 16-Item Quick Inventory of Depressive Symptomatology (QIDS), clinician rating (QIDS-C), and self-report (QIDS-SR): a psychometric evaluation in patients with chronic major depression. *Biol Psychiatry* 2003;54(5):573-83.

30. Ignatiadis M, Polyzos A, Stathopoulos GP, et al. A multicenter phase II study of docetaxel in combination with gefitinib in gemcitabine-pretreated patients with advanced/metastatic pancreatic cancer. *Oncology* 2006;71(3-4):159-63. doi: 10.1159/000106064 [published Online First: 2007/07/25]

31. Reilly TJ, MacGillivray SA, Reid IC, et al. Psychometric properties of the 16-item Quick Inventory of Depressive Symptomatology: a systematic review and meta-analysis. *J Psychiatr Res* 2015;60:132-40. doi: 10.1016/j.jpsychires.2014.09.008 [published Online First: 2014/10/11]

For peer review only - http://bmjopen.bmj.com/site/about/guidelines.xhtml

32. Gili M, Lopez-Navarro E, Homar C, et al. Psychometric properties of Spanish version of QIDS-SR16 in depressive patients. *Actas Esp Psiquiatr* 2014;42(6):292-9. [published Online First: 2014/11/13]

33. Shear MK, Vander Bilt J, Rucci P, et al. Reliability and validity of a structured interview guide for the Hamilton Anxiety Rating Scale (SIGH-A). *Depress Anxiety* 2001;13(4):166-78. [published Online First: 2001/06/20]

34. Clark DB, Donovan JE. Reliability and validity of the Hamilton Anxiety Rating Scale in an adolescent sample. *J Am Acad Child Adolesc Psychiatry* 1994;33(3):354-60. doi: 10.1097/00004583-199403000-00009 [published Online First: 1994/03/01]

35. Rolke R, Magerl W, Campbell KA, et al. Quantitative sensory testing: a comprehensive protocol for clinical trials. *Eur J Pain* 2006;10(1):77-88. doi: 10.1016/j.ejpain.2005.02.003

36. Kremeyer B, Lopera F, Cox JJ, et al. A gain-of-function mutation in TRPA1 causes familial episodic pain syndrome. *Neuron* 2010;66(5):671-80. doi: 10.1016/j.neuron.2010.04.030

37. Koltzenburg M, Lundberg LE, Torebjork HE. Dynamic and static components of mechanical hyperalgesia in human hairy skin. *Pain* 1992;51(2):207-19.

38. Adhikari K, Reales G, Smith AJ, et al. A genome-wide association study identifies multiple loci for variation in human ear morphology. *Nature communications* 2015;6:7500. doi: 10.1038/ncomms8500 [published Online First: 2015/06/25]

39. Anderson CA, Pettersson FH, Clarke GM, et al. Data quality control in genetic case-control association studies. *Nat Protoc* 2010;5(9):1564-73. doi: 10.1038/nprot.2010.116

For peer review only

40. Guo Y, He J, Zhao S, et al. Illumina human exome genotyping array clustering and quality control. *Nat Protoc* 2014;9(11):2643-62. doi: 10.1038/nprot.2014.174

41. Visscher PM, Wray NR, Zhang Q, et al. 10 Years of GWAS Discovery: Biology, Function, and Translation. *Am J Hum Genet* 2017;101(1):5-22. doi: 10.1016/j.ajhg.2017.06.005

42. TEam RC. R: A language and environment for statistical computing. Vienna, Austria2018 [Available from: https://www.R-project.org/2018.

43. Doehring A, Kusener N, Fluhr K, et al. Effect sizes in experimental pain produced by gender, genetic variants and sensitization procedures. *PLoS ONE* 2011;6(3):e17724. doi: 10.1371/journal.pone.0017724

44. Duan G, Guo S, Zhang Y, et al. The Effect of SCN9A Variation on Basal Pain Sensitivity in the General Population: An Experimental Study in Young Women. *J Pain* 2015;16(10):971-80. doi: 10.1016/j.jpain.2015.06.011

45. NM L, Lange C. The Fundamentals of Modern Statistical Genetics: Springer 2011.

46. Mallick S, Li H, Lipson M, et al. The Simons Genome Diversity Project: 300 genomes from 142 diverse populations. *Nature* 2016;538(7624):201-06. doi: 10.1038/nature18964

47. Pagani L, Lawson DJ, Jagoda E, et al. Genomic analyses inform on migration events during the peopling of Eurasia. *Nature* 2016;538(7624):238-42. doi: 10.1038/nature19792

48. Reich D, Patterson N, Campbell D, et al. Reconstructing Native American population history. *Nature* 2012;488(7411):370-4. doi: 10.1038/nature11258

49. Alexander DH, Novembre J, Lange K. Fast model-based estimation of ancestry in unrelated individuals. *Genome Res* 2009;19(9):1655-64. doi: 10.1101/gr.094052.109

For peer review only

50. Chacon-Duque JC, Adhikari K, Fuentes-Guajardo M, et al. Latin Americans show wide-spread Converso ancestry and the imprint of local Native ancestry on physical appearance. *bioRxiv* 2018 doi: 10.1101/252155

51. Adhikari K, Fuentes-Guajardo M, Quinto-Sanchez M, et al. A genome-wide association scan implicates DCHS2, RUNX2, GLI3, PAX1 and EDAR in human facial variation. *Nature communications* 2016;7:11616. doi: 10.1038/ncomms11616 [published Online First: 2016/05/20]

52. Yang J, Lee SH, Goddard ME, et al. GCTA: A Tool for Genome-wide Complex Trait Analysis. *The American Journal of Human Genetics*;88(1):76-82. doi: 10.1016/j.ajhg.2010.11.011

53. O'Connell J, Gurdasani D, Delaneau O, et al. A general approach for haplotype phasing across the full spectrum of relatedness. *PLoS Genet* 2014;10(4):e1004234. doi: 10.1371/journal.pgen.1004234

54. Howie B, Fuchsberger C, Stephens M, et al. Fast and accurate genotype imputation in genome-wide association studies through pre-phasing. *Nat Genet* 2012;44(8):955-9. doi: 10.1038/ng.2354

55. Chang CC, Chow CC, Tellier LC, et al. Second-generation PLINK: rising to the challenge of larger and richer datasets. *Gigascience* 2015;4:7. doi: 10.1186/s13742-015-0047-8

56. Price AL, Patterson NJ, Plenge RM, et al. Principal components analysis corrects for stratification in genome-wide association studies. *Nat Genet* 2006;38(8):904-9. doi: 10.1038/ng1847 [published Online First: 2006/07/25]

57. O'Reilly PF, Hoggart CJ, Pomyen Y, et al. MultiPhen: joint model of multiple phenotypes can increase discovery in GWAS. *PLoS ONE* 2012;7(5):e34861. doi: 10.1371/journal.pone.0034861

For peer review only

58. Bakshi A, Zhu Z, Vinkhuyzen AA, et al. Fast set-based association analysis using summary data from GWAS identifies novel gene loci for human complex traits. *Sci Rep* 2016;6:32894. doi: 10.1038/srep32894

59. Seldin MF. Admixture mapping as a tool in gene discovery. *Curr Opin Genet Dev* 2007;17(3):177-81. doi: 10.1016/j.gde.2007.03.002 [published Online First: 2007/05/01]

60. van Buuren S, Groothuis-Oudshoorn K. mice: Multivariate Imputation by Chained Equations in R. *Journal of Statistical Software* 2011;45(3) doi: 10.18637/jss.v045.i03

For peer review only

Table 1: Intratester reliability of sensory function protocol

	ICC(3,1)	95% CI	p-value
CDT	0.728	0.277, 0.914	0.003
WDT	0.764	0.351, 0.927	<0.0001
TSL	0.638	0.161, 0.878	0.005
HPT	0.752	0.339, 0.922	0.002
MPT	0.928	0.767, 0.979	<0.0001
WUR	0.634	0.113, 0.880	0.012
VDT	0.956	0.860, 0.987	<0.0001
PPT	0.734	0.305, 0.915	0.002
VAS	0.893	0.667, 0.970	<0.0001
Flare area	0.610	0.095, 0.869	0.015
Brush-evoked allodynia	0.756	0.365, 0.922	0.001
Punctuate hyperalgesia	0.615	0.094, 0.871	0.013
Post-sensitisation MPT	0.941	0.808, 0.983	<0.0001
Post-sensitisation HPT	0.758	0.339, 0.924	0.002

CDT: cold detection threshold, WDT: warm detection threshold, TSL: thermal sensory limen, HPT: heat pain threshold, MPT: mechanical pain threshold, WUR: wind-up ratio, VDT: vibration detection threshold, PPT: pressure pain threshold, VAS: visual analogue scale.

For peer review only

For peer review only - http://bmjopen.bmj.com/site/about/guidelines.xhtml

Figure legends

Figure 1: Study procedure. The graph details the study procedure from recruitment to data analysis. Yellow boxes represent new procedures; green boxes indicate data generation and collection; blue boxes indicate a procedural step.

BMI: body mass index; QST: quantitative sensory testing; CDT, WDT: cold, warm detection thresholds; TSL: thermal sensory limen; VDT: vibration detection threshold; HPT: heat pain threshold; MPT: mechanical pain threshold; PPT: pressure pain threshold; WUR: wind-up ratio; VAS: visual analogue scale; AITC: allyl isothiocyanate; PC: principal components; GWAS: genome-wide association study.

For peer review only

Figure 2: Method to determine area of flare, punctuate hyperalgesia and allodynia. (A) An acetate template is used to mark a star with eight spokes containing eight points at 1cm increments on the volar forearm. (B) A small cotton swab soaked in 30% mustard oil is applied in the middle of the star and (C) held in place with a tegaderm for 10 minutes. During this time, pain scores are recorded every 30 seconds. (D and E) After removal of the mustard oil, the skin flare will be marked and the area calculated. (F) The area of brush evoked and punctuate hypersensitivity will be determined with a brush and a 98.1mN von Frey hair respectively (pictured) by testing potential hypersensitivity at each point on the eight spokes.

Figure 3: Estimated power (in percentage) under the standard GWAS settings of using whole-genome genotyping data. (A) estimated power (in percentage) as a heatmap, setting

For peer review only - http://bmjopen.bmj.com/site/about/guidelines.xhtml

the significance threshold at 5×10^{-8}, the commonly used threshold for genome-wide significance in GWAS studies. (B) estimated power with the significance threshold set at 10^{-5}, the commonly used threshold for suggestive significance.

In panels A-B the X-axis denotes a range of sample sizes (n) in a GWAS, the Y-axis represents the proportion of trait variance (q^2) explained by a marker. Power of detecting the marker at a specific (n, q^2) combination is represented by a color gradient. Contour lines for power at 10% intervals are also shown.

Panels C-D shows power curves for the expected sample sizes for this study. (C) Expected power at genome-wide and suggestive significance thresholds for a sample size of n=1500. (D) Estimated power for a sample size of n=2000.

In Panels C-D the X axis denotes the proportion of trait variance (q^2) explained by a marker, and Y axis represents estimated power (in percentage). The two curves correspond to the two commonly used GWAS thresholds.

In each panel, the point for 80% power is indicated with a green triangle, so that the necessary parameter configurations can be read from the graph. In Panels A-B the contour corresponding to 80% power is also marked in green.

Figure 4: (A) Distributions of trait variance explained by a single marker from Doehring et al[43] for traits included in our study and those not included. (B) Allele frequency distributions of loci associated with experimental pain in previously published cohorts, for Europeans and Colombians.

For peer review only - http://bmjopen.bmj.com/site/about/guidelines.xhtml

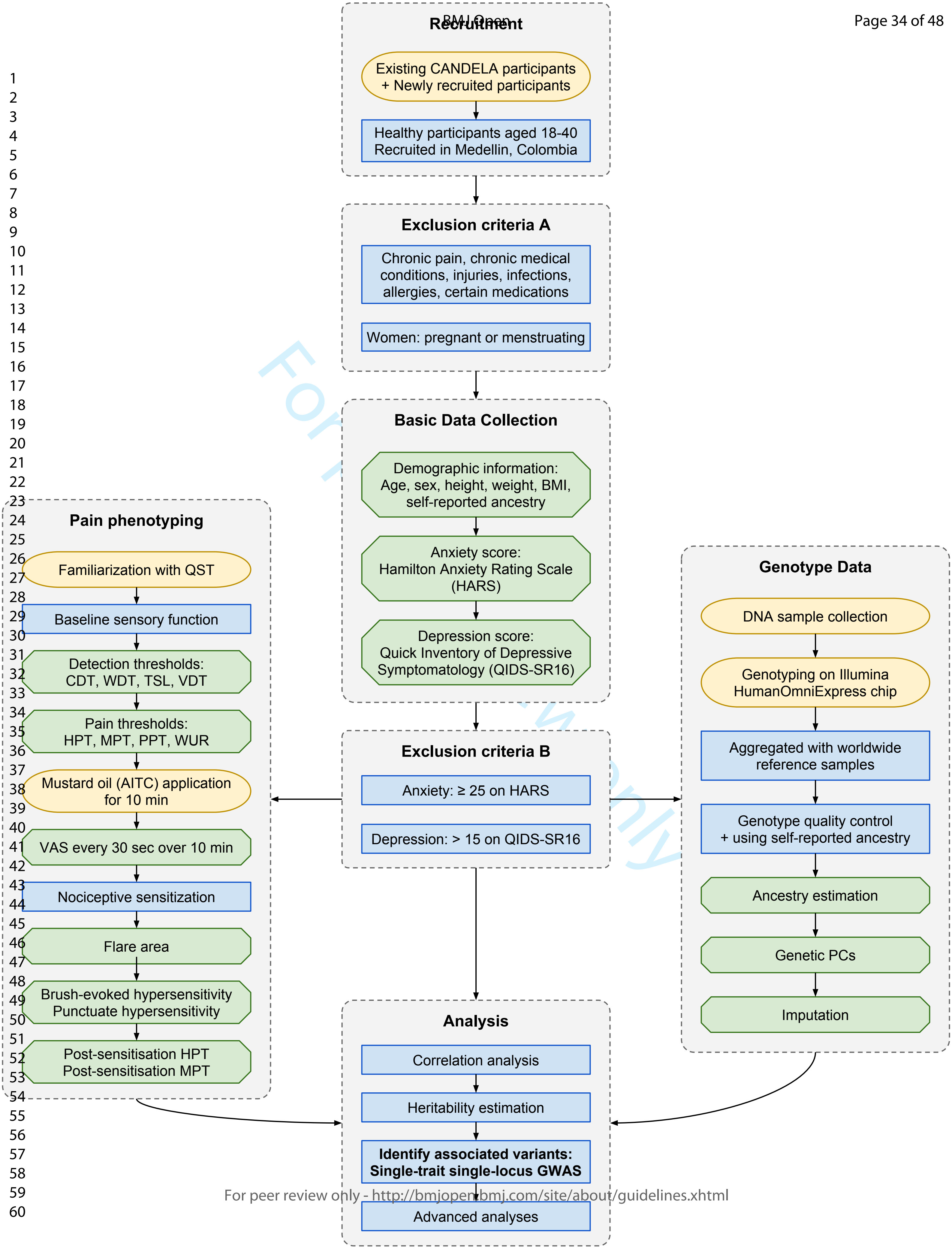
Recruitment
Existing CANDELA participants + Newly recruited participants
Healthy participants aged 18-40 Recruited in Medellin, Colombia
Exclusion criteria A
Chronic pain, chronic medical conditions, injuries, infections, allergies, certain medications
Women: pregnant or menstruating
Basic Data Collection
Demographic information: Age, sex, height, weight, BMI, self-reported ancestry
Anxiety score: Hamilton Anxiety Rating Scale (HARS)
Depression score: Quick Inventory of Depressive Symptomatology (QIDS-SR16)
Exclusion criteria B
Anxiety: ≥ 25 on HARS
Depression: > 15 on QIDS-SR16
Pain phenotyping
Familiarization with QST
Baseline sensory function
Detection thresholds: CDT, WDT, TSL, VDT
Pain thresholds: HPT, MPT, PPT, WUR
Mustard oil (AITC) application for 10 min
VAS every 30 sec over 10 min
Nociceptive sensitization
Flare area
Brush-evoked hypersensitivity Punctuate hypersensitivity
Post-sensitisation HPT Post-sensitisation MPT
Genotype Data
DNA sample collection
Genotyping on Illumina HumanOmniExpress chip
Aggregated with worldwide reference samples
Genotype quality control + using self-reported ancestry
Ancestry estimation
Genetic PCs
Imputation
Analysis
Correlation analysis
Heritability estimation
Identify associated variants: Single-trait single-locus GWAS
Advanced analyses

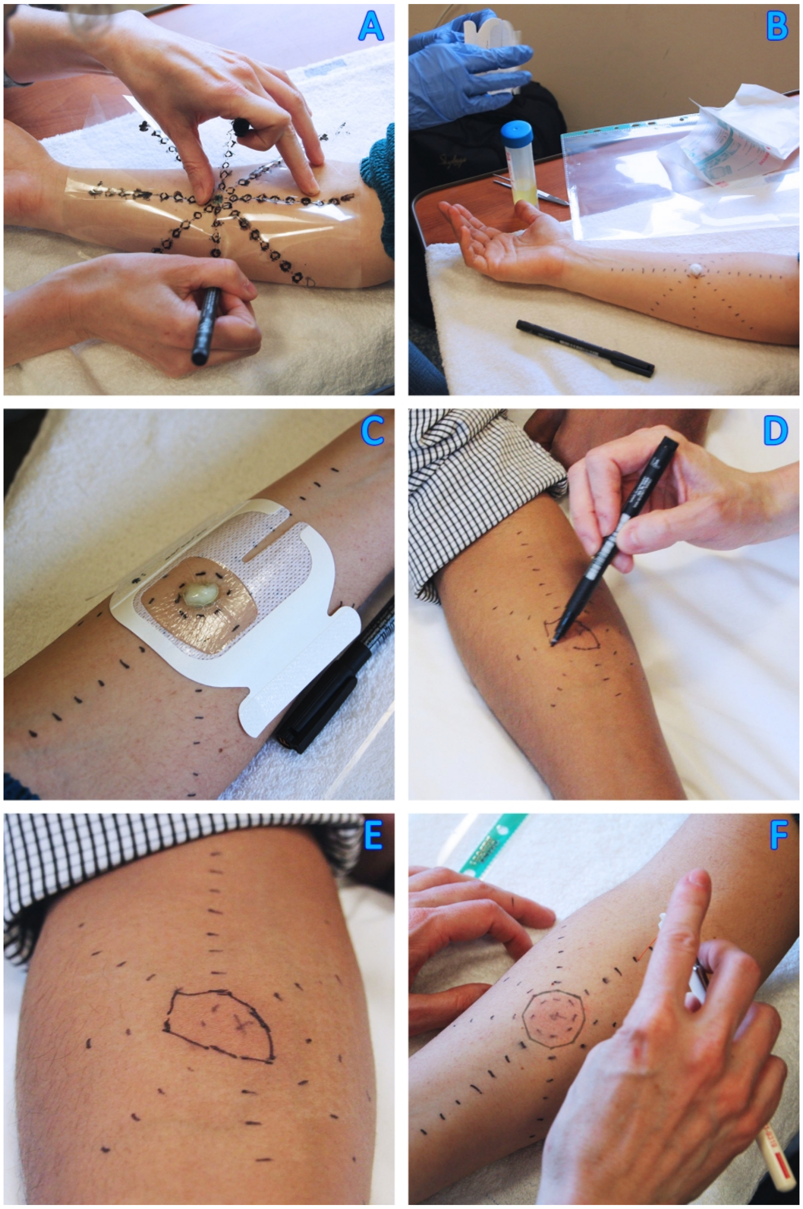

Method to determine area of flare, punctuate hyperalgesia and allodynia. (A) An acetate template is used to mark a star with eight spokes containing eight points at 1cm increments on the volar forearm. (B) A small cotton swab soaked in 30% mustard oil is applied in the middle of the star and (C) held in place with a tegaderm for 10 minutes. During this time, pain scores are recorded every 30 seconds. (D and E) After removal of the mustard oil, the skin flare will be marked and the area calculated. (F) The area of brush evoked and punctuate hypersensitivity will be determined with a brush and a 98.1mN von Frey hair respectively (pictured) by testing potential hypersensitivity at each point on the eight spokes.

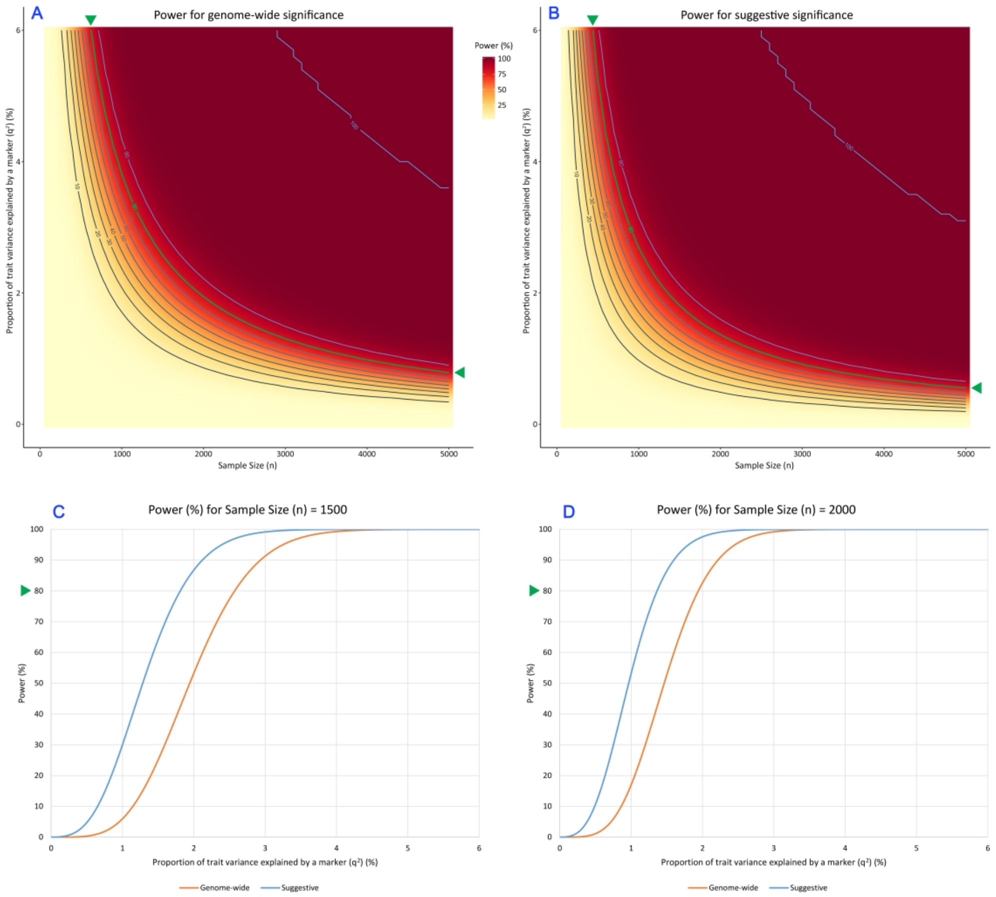

Estimated power (in percentage) under the standard GWAS settings of using whole-genome genotyping data. (A) estimated power (in percentage) as a heatmap, setting the significance threshold at 5×10-8, the commonly used threshold for genome-wide significance in GWAS studies. (B) estimated power with the significance threshold set at 10-5, the commonly used threshold for suggestive significance.

In panels A-B the X-axis denotes a range of sample sizes (n) in a GWAS, the Y-axis represents the proportion of trait variance (q2) explained by a marker. Power of detecting the marker at a specific (n, q2) combination is represented by a color gradient. Contour lines for power at 10% intervals are also shown.

Panels C-D shows power curves for the expected sample sizes for this study. (C) Expected power at genome-wide and suggestive significance thresholds for a sample size of n=1500. (D) Estimated power for a sample size of n=2000.

In Panels C-D the X axis denotes the proportion of trait variance (q2) explained by a marker, and Y axis represents estimated power (in percentage). The two curves correspond to the two commonly used GWAS thresholds.

In each panel, the point for 80% power is indicated with a green triangle, so that the necessary parameter configurations can be read from the graph. In Panels A-B the contour corresponding to 80% power is also marked in green.

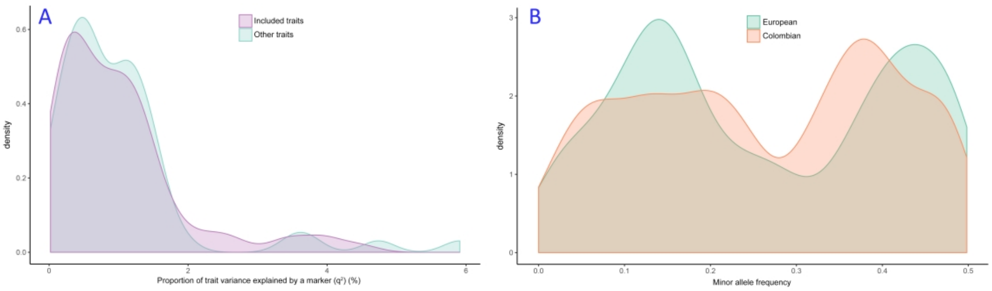

(A) Distributions of trait variance explained by a single marker from Doehring et al43 for traits included in our study and those not included. (B) Allele frequency distributions of loci associated with experimental pain in previously published cohorts, for Europeans and Colombians.

Appendix 1: Demographic and Ancestry Questionnaire

1. Age (years):

2. Gender: female (0) male (1)

3. Do you consider yourself:

Black	
Mulatto	
Indigenous	
Moreno (Brown skin)	
Cobrizo (Coppery skin)	
Mestizo (Mixed)	
Pardo (Dark skin)	
Blanco (White skin)	
European	
Other (please describe in your own words)	

4. What ancestral proportions do you think you have? And in what proportions do you consider you have each of these components?

Mark with an X the percentage range you consider to have from each ancestry. If you believe you do not bear a specific ancestry, mark the range between 0-20%.

**Indigenous**		**Black**		**European**	
0-20% (none – very low)		0-20% (none – very low)		0-20% (none – very low)	
20-40% (low)		20-40% (low)		20-40% (low)	
40-60% (medium)		40-60% (medium)		40-60% (medium)	
60-80% (high)		60-80% (high)		60-80% (high)	
80-100% (very high - full)		80-100% (very high - full)		80-100% (very high - full)	

5. If you believe that you have another ancestral component (ancestors from other races), please let us know which ones:

For peer review only

Appendix 2: Current procedures for Quality Control and Imputation in CANDELA genotype data

The currently used protocol for quality control (QC) and genotype imputation for the CANDELA genotype data is quoted from the latest GWAS publication on this cohort[1]. The entire cohort comprises samples from five Latin American countries, thus being much bigger in size and more genetically diverse; in contrast, the current experimental pain cohort is smaller and restricted to one country, so some of the specific parameter choices could be slightly different while analysing its genotype data. However, the broad procedures and most parameters should remain the same.

Genotype data

DNA samples from participants were genotyped on the Illumina HumanOmniExpress chip, which includes 730,525 SNPs. PLINK v1.9[2] was used to exclude SNPs and individuals with more than 5% missing data, markers with minor allele frequency <1%, related individuals with IBD > 0.1 (i.e. removing 3rd degree relatives and higher), and those who failed the X-chromosome sex concordance check (sex estimated from X-chromosome heterozygosity not matching recorded sex information). After applying these filters, 669,462 SNPs and 6,357 individuals were retained for further analysis.

Due to the Native American, European and African admixture of the study sample, there is inflation in Hardy-Weinberg P-values. We therefore did not exclude markers based on Hardy-Weinberg deviation, but performed stringent quality controls at software and biological levels (see also Supplementary Figure 14 from Adhikari et al.[3]). The SNP quality metrics generated from the GenCall algorithm in GenomeStudio were used for quality control. SNPs with low GenTrain score (<0.7), low Cluster Separation score (<0.3) or high heterozygosity values (|het. excess|>0.5) were excluded[4]. The heterozygosity excess filter performs a function similar to a HWE check, but is more direct since it is

For peer review only

based on the heterozygosity value, which unlike the P value does not depend on sample size. Only SNPs that satisfy these criteria across all genotyping plates were retained[3].

The imputation 'concordance' score, which is a measure of poor genotyping quality, was also used to exclude some genotyped SNPs (see below). Finally, subsequent to the GWAS analyses (see below), the genotyping cluster plots for the index SNP identified were checked manually to verify genotyping quality.

An LD-pruned set of 160,858 autosomal SNPs was used to estimate continental ancestry using the ADMIXTURE program[5]. Genetic Principal Components (PCs) were also obtained from this LD-pruned subset of SNPs. Individual outliers, including individuals with >20% African or >5% East Asian ancestry, as estimated by ADMIXTURE, were removed. Outlier individuals observed on the PC scatter plots were also removed, and PCs were recalculated each time after the removal of such individuals until no outliers remained. The number of PCs to be included in the GWAS was determined by inspecting the proportion of variance explained and by checking scree and PC scatter plots.

Genotype imputation

The chip genotype data was phased using SHAPEIT2[6]. IMPUTE2[7] was then used to impute genotypes at untyped SNPs using variant positions from the 1000 Genomes Phase 3 data. The 1000 Genomes reference data set included haplotype information for 1,092 individuals across the world for 36,820,992 variant positions.

Positions that are monomorphic in 1000 Genomes Latin American samples (Colombia, Mexico and Puerto Rico) were excluded, leading to 11,025,002 SNPs being imputed in our dataset. Of these, 48,695 had imputation quality scores < 0.4 and were excluded. Median 'info' score (imputation certainty score) provided by IMPUTE2 for the remaining imputed SNPs was 0.986. The IMPUTE2 genotype probabilities at each locus were converted into most probable genotypes using PLINK v1.9[2]

For peer review only

(at the default setting of <0.1 uncertainty). Imputed SNPs with >5% uncalled genotypes or minor allele frequency < 1% were excluded.

IMPUTE2 provides a 'concordance' metric for chip genotyped SNPs, obtained by masking the SNP genotypes and imputing it using nearby chip SNPs. Genotyped SNPs having a low concordance value (< 0.7) or a large gap between info and concordance values (info_type0 – concord_type0 > 0.1), two suggested indicators of poor genotyping quality, were also removed. Median concordance values of the remaining chip SNPs was 0.994. After QC, the final imputed dataset contained genotypes for 9,143,600 SNPs.

References:

1. Adhikari, K. et al. A GWAS in Latin Americans highlights the convergent evolution of lighter skin pigmentation in Eurasia. To appear in Nature Communications, 2019.

2. Chang, C.C. et al. Second-generation PLINK: rising to the challenge of larger and richer datasets. Gigascience 4, 7 (2015).

3. Adhikari, K. et al. A genome-wide association scan implicates DCHS2, RUNX2, GLI3, PAX1 and EDAR in human facial variation. Nat Commun 7, 11616 (2016).

4. Illumina Inc. GenomeStudio Genotyping Module v1.0 User Guide. (2008).

5. Alexander, D.H., Novembre, J. & Lange, K. Fast model-based estimation of ancestry in unrelated individuals. Genome Res 19, 1655-64 (2009).

6. O'Connell, J. et al. A general approach for haplotype phasing across the full spectrum of relatedness. PLoS Genet 10, e1004234 (2014).

7. Howie, B., Fuchsberger, C., Stephens, M., Marchini, J. & Abecasis, G.R. Fast and accurate genotype imputation in genome-wide association studies through pre-phasing. Nat Genet 44, 955-9 (2012).

For peer review only

Appendix 3: Power Calculations

The power for Genome-wide association studies of experimental pain phenotypes for varying sample and effect sizes was estimated following the formulae described in Visscher et al[1]. Estimated power is shown for a range of effect sizes for experimental pain phenotypes taken from existing experimental pain studies. The statistical software R version 3.4.1[2] was used to perform the calculations and produce the figures. The codes are published on https://github.com/kaustubhad/gwas-power.

In whole-genome SNP-based GWAS studies, the association analysis is usually conducted with a multivariate linear regression model, where the trait values are regressed onto a SNP genotype (with additive coding) and other covariates which commonly include age, gender, BMI, and genetic principal components (PCs). The p-value threshold[1,3] for genome-wide significant associations is commonly 5×10^{-8}, while the threshold for a suggestive significant association is commonly 10^{-5}.

Under the commonly used GWAS linear regression model, the term corresponding to the SNP genotype leads to a test statistic which is distributed as a chi-square distribution with 1 degrees of freedom (df). Under the null of no association it is a central chi-square, whereas under the alternative it is a non-central chi-square distribution whose non-centrality parameter (NCP) can be derived[1,4]. Power of a GWAS depends on the allele frequency of the SNPs through their effect on the NCP.

The significance threshold for the test statistic under a central chi-square (i.e. under the null) of df 1 and p-value cut-off $p = 5\times10^{-8}$ is:

$$t = F^{-1}(1-p, 1) = 29.72$$

For peer review only

Where F is the cumulative distribution function (CDF) of a central chi-square distribution, i.e. t is the (1-p)-th quantile of the distribution[4].

The power (P) of detecting an association with a trait in a GWAS, which is the probability of the observed test statistic exceeding the significance threshold t under the alternative, depends on its non-centrality parameter (λ)[4].

$$P = 1 - G(t, \lambda, 1)$$

Where G is the CDF of the non-central chi-square distribution, and df = 1 as usual.

The non-centrality parameter (λ) depends on sample size (n) and the proportion of phenotypic variance that is explained by the SNP[1], denoted by q^2.

$$\lambda = n \times \frac{q^2}{1 - q^2}$$

Thus we can vary the values of sample size (n) and the proportion of phenotypic variance explained by the SNP, denoted by (q^2), to obtain various values λ of and calculate the corresponding power (P).

These derivations include various model assumptions, e.g. the chi-square distribution assumption depends on the trait being continuous and the errors being approximately normally distributed. The assumption of normal distribution of errors might not hold in reality but in large sample sizes commonly used in GWAS they tend to hold approximately.

Similar calculations can be performed for the commonly used p-value threshold for suggestive significance, $p = 10^{-5}$. The significance threshold for the chi-square test statistic at a suggestive level is t = 19.51.

For peer review only

References:

1. Visscher PM, Wray NR, Zhang Q, et al. 10 Years of GWAS Discovery: Biology, Function, and Translation. Am J Hum Genet 2017;101(1):5-22. doi: 10.1016/j.ajhg.2017.06.005

2. R Core Team. R: A language and environment for statistical computing. Vienna, Austria2018 [Available from: https://www.R-project.org/2018.

3. Adhikari K, Reales G, Smith AJ, et al. A genome-wide association study identifies multiple loci for variation in human ear morphology. Nature communications 2015;6:7500. doi: 10.1038/ncomms8500

4. Rao CR. Linear Statistical Inference and its Applications. 2nd ed: Wiley 1973.

For peer review only

Reporting checklist for genetic association study.

Based on the STREGA guidelines.

Instructions to authors

Complete this checklist by entering the page numbers from your manuscript where readers will find each of the items listed below.

Your article may not currently address all the items on the checklist. Please modify your text to include the missing information. If you are certain that an item does not apply, please write "n/a" and provide a short explanation.

Upload your completed checklist as an extra file when you submit to a journal.

In your methods section, say that you used the STREGA reporting guidelines, and cite them as:

Little J, Higgins JP, Ioannidis JP, Moher D, Gagnon F, von Elm E, Khoury MJ, Cohen B, Davey-Smith G, Grimshaw J, Scheet P, Gwinn M, Williamson RE, Zou GY, Hutchings K, Johnson CY, Tait V, Wiens M, Golding J, van Duijn C, McLaughlin J, Paterson A, Wells G, Fortier I, Freedman M, Zecevic M, King R, Infante-Rivard C, Stewart A, Birkett N; STrengthening the REporting of Genetic Association Studies. STrengthening the REporting of Genetic Association Studies (STREGA): An Extension of the STROBE Statement.

		Reporting Item	Page Number
Title	#1a	Indicate the study’s design with a commonly used term in the title or the abstract	1 and 3
Abstract	#1b	Provide in the abstract an informative and balanced summary of what was done and what was found	3
	#2	Explain the scientific background and rationale for the investigation being reported	5-7
	#3	State specific objectives, including any prespecified hypotheses. State if the study is the first report of a genetic association, a replication effort, or both.	7
	#4	Present key elements of study design early in the paper	7-8
	#5	Describe the setting, locations, and relevant dates, including periods of recruitment, exposure, follow-up, and data collection	7-8

For peer review only

#6a	Cohort study – Give the eligibility criteria, and the sources and methods of selection of participants. Describe methods of follow-up. Case-control study – Give the eligibility criteria, and the sources and methods of case ascertainment and control selection. Give the rationale for the choice of cases and controls. Cross-sectional study – Give the eligibility criteria, and the sources and methods of selection of participants. Give information on the criteria and methods for selection of subsets of participants from a larger study, when relevant.	7-8
#6b	Cohort study – For matched studies, give matching criteria and number of exposed and unexposed. Case-control study – For matched studies, give matching criteria and the number of controls per case.	NA
#7a	Clearly define all outcomes, exposures, predictors, potential confounders, and effect modifiers. Give diagnostic criteria, if applicable	8-11
#7b	Clearly define genetic exposures (genetic variants) using a widely-used nomenclature system. Identify variables likely to be associated with population stratification (confounding by ethnic origin).	11-12
#8a	For each variable of interest give sources of data and details of methods of assessment (measurement). Describe comparability of assessment methods if there is more than one group. Give information separately for for exposed and unexposed groups if applicable.	8-11, Appendix 1
#8b	Describe laboratory methods, including source and storage of DNA, genotyping methods and platforms (including the allele calling algorithm used, and its version), error rates and call rates. State the laboratory / centre where genotyping was done. Describe comparability of laboratory methods if there is more than one group. Specify whether genotypes were assigned using all of the data from the study simultaneously or in smaller batches.	11-12
#9a	Describe any efforts to address potential sources of bias	Appendix 2
#9b	Describe any efforts to address potential sources of bias	Appendix 2
#10	Explain how the study size was arrived at	12-15, Appendix 3

For peer review only

#11	Explain how quantitative variables were handled in the analyses. If applicable, describe which groupings were chosen, and why. If applicable, describe how effects of treatment were dealt with.	15-17
#12a	Describe all statistical methods, including those used to control for confounding. State software version used and options (or settings) chosen.	15-17
#12b	Describe any methods used to examine subgroups and interactions	15-17
#12c	Explain how missing data were addressed	17
#12d	If applicable, explain how loss to follow-up was addressed	NA
#12e	Describe any sensitivity analyses	NA
#12f	State whether Hardy-Weinberg equilibrium was considered and, if so, how.	NA
#12g	Describe any methods used for inferring genotypes or haplotypes	15-17
#12h	Describe any methods used to assess or address population stratification.	NA
#12i	Describe any methods used to address multiple comparisons or to control risk of false positive findings.	15-17
#12j	Describe any methods used to address and correct for relatedness among subjects	15-17
#13a	Report numbers of individuals at each stage of study—eg numbers potentially eligible, examined for eligibility, confirmed eligible, included in the study, completing follow-up, and analysed. Give information separately for for exposed and unexposed groups if applicable. Report numbers of individuals in whom genotyping was attempted and numbers of individuals in whom genotyping was successful.	NA
#13b	Give reasons for non-participation at each stage	NA
#13c	Consider use of a flow diagram	Figure 1
#14a	Give characteristics of study participants (eg demographic, clinical, social) and information on exposures and potential confounders. Give information separately for exposed and unexposed groups if applicable. Consider giving information by genotype	NA
#14b	Indicate number of participants with missing data for each variable of interest	NA
#14c	Cohort study – Summarize follow-up time, e.g. average and total amount.	NA

For peer review only

#15	Cohort study Report numbers of outcome events or summary measures over time. Give information separately for exposed and unexposed groups if applicable. Report outcomes (phenotypes) for each genotype category over time Case-control study – Report numbers in each exposure category, or summary measures of exposure.Give information separately for cases and controls . Report numbers in each genotype category. Cross-sectional study – Report numbers of outcome events or summary measures. Give information separately for exposed and unexposed groups if applicable. Report outcomes (phenotypes) for each genotype category	NA
#16a	Give unadjusted estimates and, if applicable, confounder-adjusted estimates and their precision (eg, 95% confidence interval). Make clear which confounders were adjusted for and why they were included	NA
#16b	Report category boundaries when continuous variables were categorized	NA
#16c	If relevant, consider translating estimates of relative risk into absolute risk for a meaningful time period	NA
#16d	Report results of any adjustments for multiple comparisons	NA
#17a	Report other analyses done—e.g., analyses of subgroups and interactions, and sensitivity analyses	NA
#17b	Report other analyses done—e.g., analyses of subgroups and interactions, and sensitivity analyses	NA
#17c	Report other analyses done—e.g., analyses of subgroups and interactions, and sensitivity analyses	NA
#18	Summarise key results with reference to study objectives	NA
#19	Discuss limitations of the study, taking into account sources of potential bias or imprecision. Discuss both direction and magnitude of any potential bias.	4
#20	Give a cautious overall interpretation considering objectives, limitations, multiplicity of analyses, results from similar studies, and other relevant evidence.	18
#21	Discuss the generalisability (external validity) of the study results	NA
#22	Give the source of funding and the role of the funders for the present study and, if applicable, for the original study on which the present article is based	19

For peer review only

The STREGA checklist is distributed under the terms of the Creative Commons Attribution License CC-BY.

This checklist can be completed online using https://www.goodreports.org/, a tool made by the EQUATOR Network in collaboration with Penelope.ai

For peer review only
